# Nitrogen and phosphorus significantly alter growth, nitrogen fixation, anatoxin-a content, and the transcriptome of the bloom-forming cyanobacterium, *Dolichospermum*

**DOI:** 10.3389/fmicb.2022.955032

**Published:** 2022-09-07

**Authors:** Benjamin J. Kramer, Jennifer G. Jankowiak, Deepak Nanjappa, Matthew J. Harke, Christopher J. Gobler

**Affiliations:** ^1^School of Marine and Atmospheric Sciences, Stony Brook University, Southampton, NY, United States; ^2^Jonah Ventures, Boulder, CO, United States; ^3^Gloucester Marine Genomics Institute, Gloucester, MA, United States

**Keywords:** nitrogen, ammonium, nitrate, urea, phosphorus, N_2_ fixation, *Dolichospermum*, anatoxin-a

## Abstract

While freshwater cyanobacteria are traditionally thought to be limited by the availability of phosphorus (P), fixed nitrogen (N) supply can promote the growth and/or toxin production of some genera. This study characterizes how growth on N_2_ (control), nitrate (NO_3_^–^), ammonium (NH_4_^+^), and urea as well as P limitation altered the growth, toxin production, N_2_ fixation, and gene expression of an anatoxin-a (ATX-A) – producing strain of *Dolichospermum* sp. 54. The transcriptomes of fixed N and P-limited cultures differed significantly from those of fixed N-deplete, P-replete (control) cultures, while the transcriptomes of P-replete cultures amended with either NH_4_^+^ or NO_3_^–^ were not significantly different relative to those of the control. Growth rates of *Dolichospermum* (sp. 54) were significantly higher when grown on fixed N relative to without fixed N; growth on NH_4_^+^ was also significantly greater than growth on NO_3_^–^. NH_4_^+^ and urea significantly lowered N_2_ fixation and *nifD* gene transcript abundance relative to the control while cultures amended with NO_3_^–^ exhibited N_2_ fixation and *nifD* gene transcript abundance that was not different from the control. Cultures grown on NH_4_^+^ exhibited the lowest ATX-A content per cell and lower transcript abundance of genes associated ATX-A synthesis (*ana*), while the abundance of transcripts of several *ana* genes were highest under fixed N and P - limited conditions. The significant negative correlation between growth rate and cellular anatoxin quota as well as the significantly higher number of transcripts of *ana* genes in cultures deprived of fixed N and P relative to P-replete cultures amended with NH_4_^+^ suggests ATX-A was being actively synthesized under P limitation. Collectively, these findings indicate that management strategies that do not regulate fixed N loading will leave eutrophic water bodies vulnerable to more intense and toxic (due to increased biomass) blooms of *Dolichospermum*.

## Introduction

*Dolichospermum*, formerly known as *Anabaena* (Wacklin et al., 2009), is a cyanobacterial genus that includes toxin-producing taxa which can form harmful blooms (CHABs) in fresh (O’Neil et al., 2012; Li et al., 2016) and brackish water (Paul et al., 2016; Olofsson et al., 2020) ecosystems. Blooms of this genus are increasing in frequency, intensity, and geographic range due to climate change and eutrophication (O’Neil et al., 2012; Salmaso et al., 2015; Li et al., 2016). This genus belongs to the order Nostocales (Fogg, 1942; Issa et al., 2014), a group of dinitrogen (N_2_) - fixing cyanobacteria that develops heterocysts when fixed N is limiting (Mickelson et al., 1967; El-Shehawy and Kleiner, 2003) and utilizes the enzyme nitrogenase to convert N_2_ to ammonia (Wolk et al., 1994; Hoffman et al., 2014).

Primary productivity in freshwater ecosystems is traditionally considered phosphorus (P) - limited (Correll, 1999; Blomqvist et al., 2004) because N_2_-fixing cyanobacteria, or diazotrophs, which bloom under P-replete conditions (Gobler et al., 2016; Wang et al., 2021), produce newly fixed N to support phytoplankton growth (Smith, 1983, 2016; Schindler et al., 2008). The proportion of newly fixed N relative to total nitrogen in eutrophic lakes, however, can be extremely variable (6–80%), and is often regarded as insufficient to supply CHAB taxa with N sufficient to achieve optimal biomass (Howarth et al., 1988; Scott and McCarthy, 2010; Hellweger et al., 2016) due to micronutrient and light limitation (Wurtsbaugh and Horne, 1983; Lewis and Levine, 1984) as well as denitrification (Paerl, 2017). *Dolichospermum* populations do not necessarily bloom in response to P-enrichment (Paerl et al., 2014), and in some cases exhibit a higher demand for P than non-diazotrophic cyanobacteria (Wan et al., 2019). Collectively, these data suggest that P-enrichment alone is insufficient for *Dolichospermum* to achieve maximum growth rates, and that diazotrophy may not fully meet the fixed N demands required for Nostocales to bloom (Dolman et al., 2012; Shatwell and Köhler, 2018; Chaffin et al., 2019; Gamez et al., 2019). Furthermore, the fixed N species preferred by *Dolichospermum* to achieve optimal growth rates and how members of this genus respond to fixed N and P– depletion are poorly understood and warrant further investigation.

Nostocales also significantly reduce nitrogenase activity and upregulate metabolic pathways for fixed N assimilation when certain fixed N compounds are abundant, though the degree of reduced N_2_ fixation is dependent on the fixed N species provided and not always associated with enhanced growth (Sanz et al., 1995; Mekonnen et al., 2002; Zulkefli and Hwang, 2020). The effects of fixed N deprivation on *Dolichospermum* genome expression have been studied, with genes associated with heterocyst development and N_2_ fixation being significantly upregulated, for instance (Ehira et al., 2003; Flaherty et al., 2011; He et al., 2021). However, studies that have described how the *Dolichospermum* genome responds to different fixed N species (NH_4_^+^, NO_3_^–^) have never included urea, which can significantly suppress N_2_ fixation and enhance growth rates and toxin production (Ge et al., 1990; Qian et al., 2017). Since the 1980s, urea has also become the predominant fixed N fertilizer in the United States (Paerl et al., 2016), and its use in China is significantly positively correlated with the duration and number of CHABs (Glibert et al., 2014). The effects of P depletion on *Dolichospermum* genome expression availability (Teikari et al., 2015) have also never been coupled to changes in fixed N availability and would be essential for understanding how co-limitation affects this genus.

*Dolichospermum* spp. can produce a wide variety of toxins, including the neurotoxic congener anatoxin-a (ATX-A) (Devlin et al., 1977; Sanchez et al., 2014; Christensen and Kahn, 2020). ATX-A mimics the action of the neurotransmitter acetylcholine (Carmichael et al., 1979; Spivak et al., 1980), causing muscle fatigue, paralysis, or even death (Carmichael, 1994; Humpage et al., 1994; Lilleheil et al., 1997), and is synthesized by enzymes encoded by genes belonging to the anatoxin synthetase gene (*ana*) cluster (Rantala-Ylinen et al., 2011). ATX-A - producing *Dolichospermum* strains originate from several continents, including North America, Europe, and Australia (John et al., 2019; Österholm et al., 2020). While ATX-A concentrations and the growth of some Nostocales strains can significantly increase in response to fixed N enrichment (Dolman et al., 2012; Qian et al., 2017) and a higher dissolved N:P ratio (Toporowska et al., 2016), other strains exhibit significantly reduced ATX-A quotas but significantly higher biomass in response to NO_3_^–^ amendment (Gagnon and Pick, 2012). Thus, the effects of different fixed N forms and P-limitation on *Dolichospermum* ATX-A production are unclear and warrant further investigation.

Ultimately, while *Dolichospermum* growth and N_2_ fixation rates can be responsive to fixed N, the differential effects of different fixed N species as well as fixed N and P co-limitation on members of this genus have not been investigated. Thus, the objective of this study was to quantify the effects of NH_4_^+^, NO_3_^–^, and urea, as well as fixed N and P co-limitation, on the growth, N_2_ fixation, photosystem II photosynthetic efficiency (*F_*v*_/F_*m*_*), toxin production, and genome-wide expression of *Dolichospermum* sp. 54, an ATX-A-producing taxon. We hypothesized that the availability of any of the three fixed N forms would lead to significant reductions in N_2_ fixation and increases in growth, while ATX-A cellular quotas would be directly controlled by the growth rates of *Dolichospermum*. These findings will likely be useful for predicting how CHABs dominated by *Dolichospermum* and ATX-A production respond to differing nutrient regimes.

## Materials and methods

### Experimental design

*Dolichospermum* sp. strain 54 (ATX-A producer) is a non-axenic isolate from a southern Finnish lake (Rouhiainen et al., 1995) that was grown in batch cultures in the freshwater medium BG11 (Stainer et al., 1971) without fixed N (BG11-N) for several weeks prior to experimentation to promote N_2_ fixation. Immediately prior to experimentation, cells from these cultures, which were in early stationary phase, were enumerated (see below) and centrifuged at 3000 RCF for 10 min, the supernatant removed, and cell pellets resuspended in BG11-N to avoid changing the target fixed N concentrations in experimental treatments. 500 mL Erlenmeyer flasks containing 300 mL BG11 modified with different forms and concentrations of fixed N or without P were then inoculated with the concentrated *Dolichospermum* sp. 54 sample, and used for five experimental nutrient treatments, each with three replicates (*n* = 3) having an initial culture concentration of approximately 5 × 10^5^ cells mL^–1^. Target cell densities were determined from the concentrated sample (C_1_V_1_ = C_2_V_2_), and fixed N was added post-inoculation. The five experimental treatments were: BG11 with ammonium chloride (NH_4_^+^ + P = 100 μM), urea (urea + P = 50 μM), or NaNO_3_ (NO_3_^–^ + P = 100 μM) as nitrogen forms, BG11-N (-N + P, or the control), and BG11 without fixed nitrogen or phosphorus (-N-P). Potassium hydrogen phosphate concentrations (K_2_HPO_4_) were kept the same in all treatments receiving P (0.23 mM). Flasks were then placed in an incubator set at 21°C on a 14:10 light/dark cycle at ∼40 μmol photons m^–2^ s^–1^ and bubbled with ambient air passed through 0.2 μm HEPA filters at a rate of ∼200 mL min^–1^. Sample processing and culture maintenance began in the late morning (10:00 – 11:00) during the experiment. Cultures treated with fixed nitrogen were amended again with the same nitrogen species at the same starting concentration on days 3 through 6 of the 9-day experiment to ensure that the fixed N pool did not become depleted. While the starting dissolved N:P ratios of these treatments were less than that of Redfield (1958) in fixed N-amended treatments, concentrations of NO_3_^–^ and NH_4_^+^ were kept the same to assure comparability between treatments and were kept relatively low (μM), as both NH_4_^+^ (Dai et al., 2008) and urea (Sakamoto et al., 1998) are toxic to cyanobacteria at high (mM) concentrations. Every day and prior to the final time point (day 9), 10 mL of sample was removed from each flask for cell density enumeration (5 mL) and for measuring photosynthetic efficiency (5 mL); an extra 6 mL was used to measure N_2_ fixation (5 mL) and alkaline phosphatase activity (1 mL). The experiment concluded after cultures had been in the exponential phase of growth for several days, differences in cell densities between treatments were apparent (Supplementary Figure 1), and −N-P cultures displayed signs of P-limitation (i.e., elevated alkaline phosphatase activity relative to the control and lower *F_*v*_/F_*m*_* relative to fixed N - amended cultures; see below).

### Culture analyses

Cells were preserved in Lugol’s iodine each day then enumerated under an inverted Nikon Eclipse TS100 microscope using a gridded 1 mm^2^ Sedgewick Rafter counting chamber. Cell densities, which exhibited exponential growth as the experiment progressed (Supplementary Figure 1), were then used to determine maximum growth rates (μ_max_), measured as *day*^−1^ = (LN(N/N))/(t−t), where the difference in cell density (N) between days *t*_2_ and *t*_1_ represents the greatest (positive) change in cell density throughout the duration of the experiment (Guillard, 1973). At specific time points, cell density (cells mL^–1^) was normalized to both ATX-A concentrations (μg L^–1^) and the amount of N_2_ fixed (μmol N_2_-fixed L^–1^ day^–1^) to determine cellular quotas of ATX-A and N_2_ fixation rates per cell.

Dissolved fixed N samples were collected by passing sample through a pre-combusted (450°C) glass fiber filter (GF/F) and stored frozen (-20°C) until analysis on a Lachat Instruments autosampler (ASX-520). Specifically, nitrate was analyzed using the Greiss reaction following reduction of NO_3_^–^ to NO_2_^–^ using cadmium columns (Jones, 1984), while ammonium was analyzed using a revised version of the indophenol blue method (Parsons et al., 1984). NO_3_^–^ and NH_4_^+^ analyses proceeded when the recovery of standard reference material (SPEX CertiPrep™) was at least 90 ± 10%. To measure *F_*v*_/F_*m*_*, or the maximum quantum efficiency of photosystem (PS) II, dark-adapted *in vivo* (F_0_) and DCMU (3,4-dichlorophenyl-1,1-dimethylurea)-enhanced *in vivo* fluorescence (F_*m*_) was measured daily on a Turner Designs TD-700 fluorometer (Ex/Em = 340 – 500/>665 nm). Readings were blank-corrected using standard BG11 media (Harke and Gobler, 2013). DCMU blocks electron transfer between photosystems, stopping photosynthesis, and is a reliable indicator of stress in eukaryotic phytoplankton and cyanobacteria due to fixed N and/or P depletion (Parkhill et al., 2001; Simis et al., 2012). Samples were also collected from −N + P and −N-P cultures to measure alkaline phosphatase activity (APA), which is a reliable indicator of stress induced by the lack of PO_4_^3–^ in aquatic systems (Dyhrman et al., 2007), every other day. APA was measured on a Turner Designs TD-700 fluorometer (Ex/Em = 300 – 400/410 – 600 nm) using 4-methylumbelliferone phosphate (250 μM) as the substrate (Hoppe, 1983). APA measured using this method correlates significantly with the expression of the gene *phoX* that encodes for alkaline phosphatase in cyanobacteria (Harke et al., 2012).

N_2_ fixation rates were measured every other day using the acetylene reduction method (Capone, 1993). Briefly, acetylene (C_2_H_2_) is reduced by nitrogenase to ethylene, as one molecule of N_2_ is fixed for every four C_2_H_4_ molecules produced in *Dolichospermum* (Hardy et al., 1973; Jensen and Cox, 1983). Acetylene was made by reacting 7 grams of lab-grade calcium carbide (Fisher Scientific) with 700 mL of deionized water (Hyman and Arp, 1987; Beversdorf et al., 2013), with the resulting gas collected in Supelco Tedlar bags. The amount of ethylene produced was quantified using C_2_H_4_ standards made by injecting 1% ethylene in N_2_ (Airgas) into air-tight vials immediately prior to sample analysis. Acetylene was injected into the headspace of air-tight 10 mL vials containing samples, which were then placed into the incubator with experimental cultures for 3-4 h, after which a portion of the headspace was extracted and injected into a Trace 1310 Gas Chromatograph (Thermo Scientific). The amount of ethylene produced was determined using Chromeleon Chromatography Data System (CDS) software (Version 7.3).

For the final timepoint (day 9) of the experiment, 25 mL of sample from each flask was collected for anatoxin analysis by filtering water through a pre-combusted GF/F and stored frozen at −20°C. ATX-A was extracted using an acetonitrile:H_2_O:formic acid (80:19.9:0.1) mixture (Dell’Aversano et al., 2005). Extracts were then quantified via liquid chromatography tandem mass spectrometry (LC-MS/MS) using an Agilent 1200 series HPLC and Agilent 6410 triple quadrupole mass spectrometer equipped with a Peak Nitrogen generator #NM30LA (Peak Scientific, Inc. Billerica, MA). ATX-A was detected using qualifier and quantifier ions (Buckland et al., 2005; James et al., 2005) using Agilent MassHunter Qualitative Analysis software (version B.03.01) and quantified by normalization to the parent compound.

### RNA isolation and sequencing

To characterize differential expression of the *Dolichospermum* sp. 54 transcriptome relative to the control, RNA was acquired using Millipore Sterivex filters (0.22 μm) at the end of the experiment (day 9), filtering 50 mL from each replicate per treatment (*n* = 3). After filtration, samples were flash frozen in liquid N_2_ and stored at −80°C. Total nucleic acids were extracted using the cetyltrimethyl ammonium bromide (CTAB) method (Dempster et al., 1999; Harke and Gobler, 2013). Briefly, 1 mL of CTAB lysis buffer was added to each frozen sample while still in the Sterivex filter, heated to 50°C, and returned to −80°C overnight. After placing frozen samples in a water bath set at 65°C, 750 μL of CTAB was carefully removed from each Sterivex filter using a syringe and transferred to an Eppendorf tube, centrifuged, extracted with 750 μL chloroform, precipitated with an isopropanol/sodium chloride solution, and centrifuged one final time.

The quality and quantity of sample nucleic acids were assessed using a Nanodrop spectrophotometer and a Qubit fluorometer in tandem with a dsDNA BR Assay kit (ThermoFisher Scientific) following the manufacturer’s instructions, then stored at −80°C. Total nucleic acid samples were treated with RNase-free DNase (Qiagen) to digest genomic DNA, and resulting total RNA was quantified using a Qubit? fluorometer and quality assessed using an Agilent QC Bioanalyzer. A relatively low amount of RNA was extracted from one of the −N-P replicates (∼3 ng μL^–1^) and was thus removed from analysis. Samples were then sent to Columbia University’s Next Generation Sequencing facility for sequencing on an Illumina HiSeq 4000 system, prior to which ribosomal RNA was depleted and sequencing libraries were prepared using a TruSeq Ribo-Zero Gold kit. 20 × 10^6^ raw 100 bp single-end reads were sequenced per sample.

### Read mapping and analysis

The quality of the raw reads was assessed using FASTQC (version 0.11.5). Through Trimmomatic (version 0.39), quality trimming was conducted on raw reads to remove adapter sequences and poor quality bases (Bolger et al., 2014). Trimming parameters were set as follows: SLIDINGWINDOW:4:15 − ILLUMINACLIP:adaptor_truseq.fasta:2:30:15 − MINLEN:30, with a custom made truseq adapter file. Cleaned, filtered, and trimmed reads were then assembled using Trinity (version v2.8.4) (Grabherr et al., 2011; Haas et al., 2013); the transcriptome assembly was evaluated using RNA-Seq. Clean reads from different treatments were first pooled and normalized using Trinity’s *in silico* normalization module, then assembled *de novo* into transcripts using Trinity’s single-end mode with default settings. The candidate open reading frames (ORFs) and deduced amino acid sequences were obtained using TransDecoder (version 5.5.0^1^) using search results of the BLASTP (BLAST + version 2.9.0) and Hmmscan (HMMER version 3.2.1) databases. Diamond BLASTX was used to annotate transcripts against the UniProt_Swiss-Prot database (release 2020.01), using the genome of *Anabaena* sp. WA102 as a reference, and an e-value of 1e^–3^ to avoid getting low annotations and mismatches.

The Kyoto Encyclopedia of Genes and Genomes (KEGG) was used to obtain KEGG orthology (KO) gene annotations from the UniProt_Swiss-Prot database. KEGG annotations were also obtained through the GHOSTX (Suzuki et al., 2014) search of KOALA (KEGG Orthology And Links Annotation) (Kanehisa et al., 2014). Both sets of KEGG IDs were then merged to get the maximum number of gene annotations. Read counts of transcripts were obtained with Kallisto (version 0.46.0) using default settings (Bray et al., 2016) by mapping the clean reads of each sample to the assembled transcriptome, and the transcript abundances were normalized to transcripts per million (TPM) values. In Sleuth (version 0.30.0), Kallisto counts were used to characterize differential gene expression, then aggregate normalized counts by KO ID (Pimentel et al., 2017). Gene transcripts were quantified using units of scaled reads per base, and Wald tests were performed in Sleuth to determine whether the experimental groups exhibited significant (false discovery rate/*qval* < 0.05) differential gene expression relative to the control (-N + P), which was determined based on beta (b) – values generated in Sleuth that represent biased estimators of log_2_-fold changes (Δ) in transcript abundance. A gene ontology (GO) enrichment analysis was also performed on the Kallisto counts to map transcripts to genes associated with certain biological, cellular, and molecular categories of genes associated with N and P metabolism, growth, and photosynthesis. Differential expression at the transcript level for each treatment relative to the control was first determined using Sleuth, then processed and mapped to gene categories using the GOStats (version 2.56.0) package (Falcon and Gentleman, 2007).

To characterize differential expression of genes associated with anatoxin synthesis, Trimmomatic reads were assembled into transcripts using SPAdes (version 3.11.1) (Bankevich et al., 2013; Nurk et al., 2013; Bushmanova et al., 2019) and annotated using Prokka (version 1.12^2^) (Seeman, 2014). The annotated assembly and the *Anabaena* sp. 37 ATX-A - encoding gene sequences available on NCBI (Rantala-Ylinen et al., 2011), were then put through BLASTP to find which sequences in the assembly matched the genes belonging to the anatoxin synthetase gene (*ana*) cluster.

### Statistical analyses and data visualization

Statistical analyses were done in R (Version 4.0.3) and graphing was done in both R and Microsoft Excel (Version 16.51). *F_*v*_/F_*m*_* and N_2_ fixation rate values for each replicate at each time point were averaged prior to being averaged with the values of other replicates from the same treatment. Using the dplyr package, a two-way analysis of variance (ANOVA) was performed to determine whether fixed N and P availability significantly affected growth rate, *F_*v*_/F_*m*_*, dissolved nutrient levels, ATX-A cell quota, N_2_ fixation rate, and normalized counts of *ana* transcripts. Tukey’s HSD tests using the agricolae package were then performed *post hoc* to determine whether differences among treatments were significantly different to each other. For two-way ANOVAs, the Shapiro-Wilk and Fligner-Killeen tests were used to confirm that the data passed normality and homogeneity of variance, respectively. Transcript data for *anaE* and *anaF* exhibited non-normal distribution under Shapiro-Wilk but did exhibit normality (*p* > 0.05) using Kolmogorov-Smirnov (KS) non-parametric tests. Significant differences in APA activity were compared between the control (-N + P) and -N-P across time using a Student’s paired *t*-test. Linear regression analyses (F-tests) were performed using the stats package to determine whether there were correlations between N_2_ fixation and growth rate, and to determine whether ATX-A content covaried with nitrogenase activity and growth rate. In all cases, an alpha level of 0.05 was utilized.

To visualize the overall expression of genomes among treatments, a non-metric multidimensional scaling (nMDS) plot of TPM values was created using the vegan package (Version 2.5-7^3^). To test for statistically significant differences between treatments, 95% confidence intervals (CIs) were drawn around experimental groups in the nMDS plot. Furthermore, a two-way permutational multivariate analysis of variance (PERMANOVA) was performed on a Bray-Curtis dissimilarity matrix using the adonis2 package to determine whether N and/or P availability significantly influenced global gene expression. As stated before (see *Read mapping and analysis*), Wald tests were also performed using the sleuth package to determine whether the experimental groups exhibited significant (false discovery rate/*qval* < 0.05) differential gene expression relative to the control (-N + P). Global results in differential expression relative to the control were visualized using Bland-Altman (MA) plots, with b-values, or log_2_-fold changes (Δ) in expression, plotted as a function of mean abundance of annotated gene transcripts. The data were further examined for genes associated with nitrogen metabolism/fixation and phosphorus assimilation using the software Morpheus (Broad Institute^4^) to generate heatmaps. Hypergeometric distribution tests (*padj* < 0.05) were performed using the GOstats package to determine whether gene categories associated with growth, N and P assimilation, and photosynthesis were significantly enriched relative to the control.

## Results

### Growth rate, nutrient availability, and physiological indicators of nutrient acquisition and stress

**FIGURE 1:**
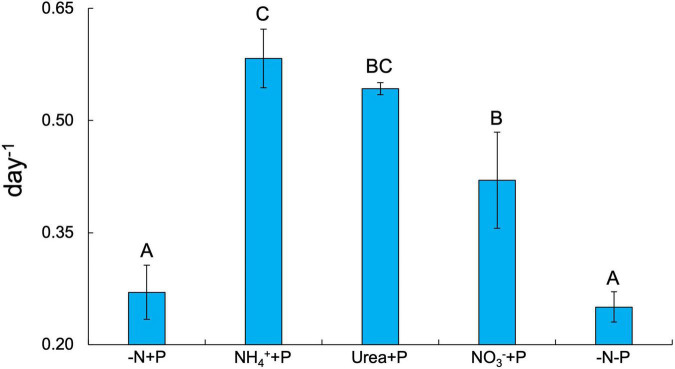
Maximum growth rates (μ_max_; day^–1^) across all experimental groups. Error bars represent standard deviation. Letters above bars represent significant differences between experimental groups (Two-way ANOVA; Tukey’s HSD *post hoc*; *p* < 0.05).

**FIGURE 2:**
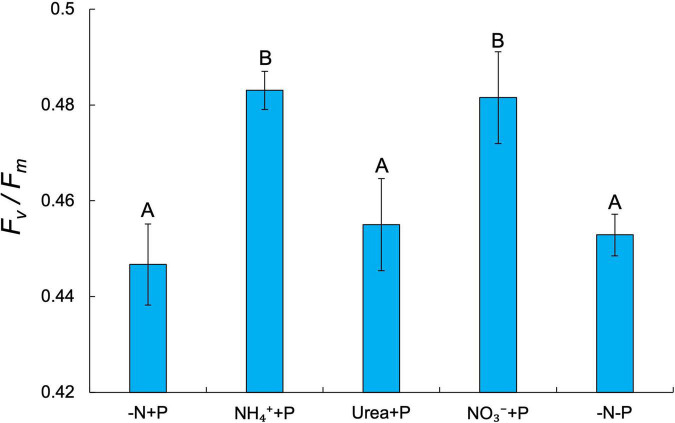
Photosystem II photosynthetic efficiency (*F_*v*_/F_*m*_*) across treatments. Error bars represent standard deviation. Letters above bars represent significant differences between experimental groups (Two-way ANOVA; Tukey’s HSD *post hoc*; *p* < 0.05).

Fixed N availability alone had a significant positive effect on the maximum growth rates (μ_*max*_ = day^–1^) of *Dolichospermum* sp. 54 (Two-way ANOVA; *p* < 0.001), as cultures amended with NH_4_^+^ (0.58 ± 0.04 day^–1^), urea (0.54 ± 0.01 day^–1^), and NO_3_^–^ (0.42 ± 0.06 day^–1^) all exhibited significantly higher μ_*max*_ than those of the control (-N + P; 0.27 ± 0.04 day^–1^) and −N-P (0.25 ± 0.02 day^–1^) cultures (Tukey’s HSD; *p* < 0.05; Figure 1). NH_4_^+^ + P cultures exhibited significantly faster growth rates relative to NO_3_^–^ + P cultures (Tukey’s HSD; *p* < 0.05; Figure 1). Dissolved NO_3_^–^ and NH_4_^+^ concentrations on days 6 and 7 of the experiment affirmed that the growth rates of fixed N-amended cultures were not limited by fixed N supply (Supplementary Table 1). PS-II photosynthetic efficiency (*F_*v*_/F_*m*_*) was significantly altered by the availability of fixed N (Two-way ANOVA; *p* < 0.001), but not P, though only two of the fixed N-enriched treatments, NH_4_^+^ + P (0.48 ± 0.004 day^–1^) and NO_3_^–^ + P (0.48 ± 0.01 day^–1^), exhibited significantly (Tukey’s HSD; *p* < 0.05) higher average *F_*v*_/F_*m*_* values than −N + P cultures (0.45 ± 0.01; Figure 2). Furthermore, on days 3-8 of the experiment, *F_*v*_/F_*m*_* values of NH_4_^+^ + P and NO_3_^–^ + P were higher than those of urea + P cultures (Supplementary Figure 2). −N-P cultures exhibited significantly (Student’s paired *t*-test; *p* < 0.05) greater APA activities relative to the control during all time points measured (Supplementary Figure 3).

### N_2_ fixation rates

**FIGURE 3:**
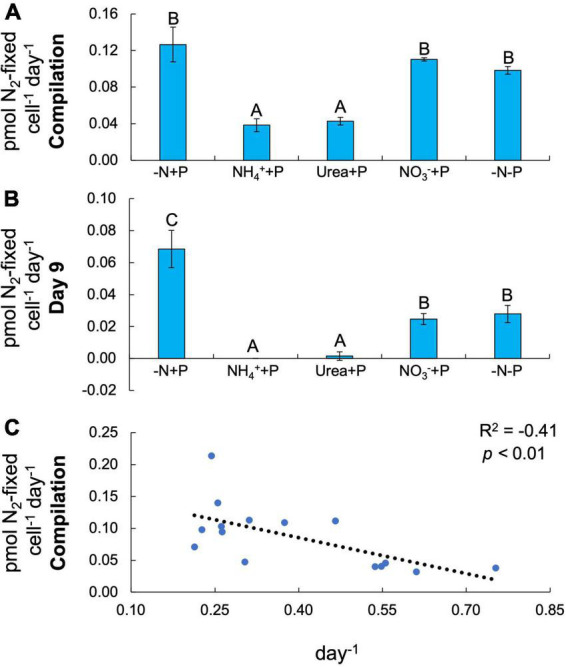
N_2_ fixation rates normalized to cell density (pmol N_2_-fixed cell^–1^ day^–1^). **(A)** N_2_ fixation rates on days 1, 3, 5, and 9 averaged. **(B)** Final timepoint (day 9) N_2_ fixation rates. Letters above bars represent significant differences between experimental groups (Two-way ANOVA; Tukey’s HSD *post hoc*; *p* < 0.05). **(C)** N_2_ fixation as a function of maximum growth rate (μ_*max*_; day^–1^). Values in the upper right-hand corner represent the coefficient of determination (R^2^) and the significance (*p*-value) of the linear regression.

Both fixed N (Two-way ANOVA; *p* < 0.001) and P (*p* < 0.01) availability significantly altered N_2_ fixation rates in *Dolichospermum* sp. 54 (Figure 3A). Amendments with reduced N compounds significantly decreased (Tukey’s HSD; *p* < 0.05) the average N_2_ fixation rate (NH_4_^+^ + P = 0.04 ± 0.01 pmol N_2_-fixed cell^–1^ day^–1^; urea + P = 0.04 ± 0.004 pmol N_2_-fixed cell^–1^ day^–1^) relative to that of control cultures (-N + P; 0.13 ± 0.02 pmol N_2_-fixed cell^–1^ day^–1^; Figure 3A). On the final day of the experiment, NO_3_^–^ + P (0.02 ± 0.003 pmol N_2_-fixed cell^–1^ day^–1^) and −N-P (0.03 ± 0.01 pmol N_2_-fixed cell^–1^ day^–1^) cultures exhibited significantly (Tukey’s HSD; *p* < 0.05) lower N_2_ fixation rates relative to the control (0.07 ± 0.01 pmol N_2_-fixed cell^–1^ day^–1^) and significantly higher N_2_ fixation rates relative to NH_4_^+^ + P (below detection limit) and urea + P (0.002 ± 0.003 pmol N_2_-fixed cell^–1^ day^–1^) cultures (Figure 3B). Across all cultures, there was a significant negative correlation between N_2_ fixation and growth rates (F-test; R^2^ = −0.41, *p* < 0.01; Figure 3C).

### Anatoxin-a cell quotas

**FIGURE 4:**
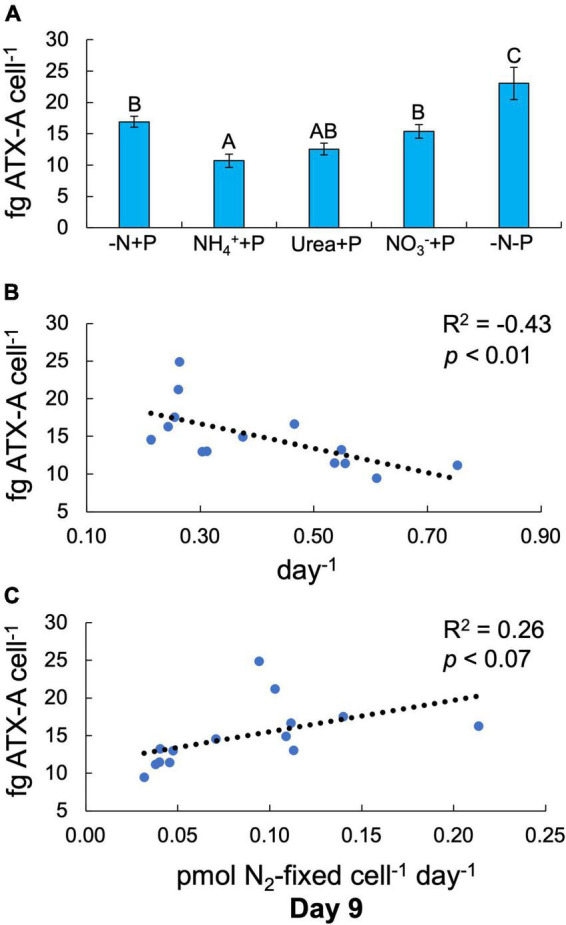
Anatoxin-a (ATX-A) content per cell (fg ATX-A cell^–1^) across treatments at the final timepoint [day 9; **(A)**]. Error bars represent standard deviation. Letters above bars represent significant differences between experimental groups (Two-way ANOVA; Tukey’s HSD *post hoc*; *p* < 0.05). Anatoxin-a (ATX-A) content per cell as functions of maximum growth rate [**(B)**; μ_*max*_; day^–1^] and N_2_ fixation **(C)**. Values in the upper right-hand corners represent coefficients of determination (R^2^) and the significance (*p*-value) of linear regressions.

Fixed N (Two-way ANOVA; *p* < 0.001) and P (*p* < 0.01) availability both significantly altered cellular ATX-A content (Figure 4). Of all experimental treatments, only NH_4_^+^ + P and −N-P exhibited significantly lower (10.7 ± 1.06 fg ATX-A cell^–1^) and higher (23.0 ± 2.59 fg ATX-A cell^–1^) cellular ATX-A quotas than the control (-N + P; 16.9 ± 0.88 ATX-A fg cell^–1^), respectively (Tukey’s HSD; *p* < 0.05; Figure 4A). ATX-A content was significantly (F-test; R^2^ = −0.43, *p* < 0.01; Figure 4B) negatively correlated with growth rate, while ATX-A content exhibited a positive, marginally significant (F-test; R^2^ = 0.26, *p* < 0.07; Figure 4C) correlation with N_2_ fixation rate.

### Global transcriptomic changes

**FIGURE 5:**
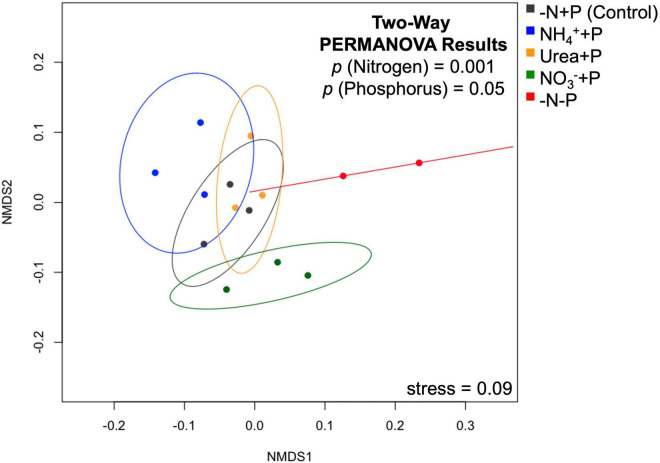
Non-metric multi-dimensional scaling (nMDS) plot of normalized read counts among treatments mapped to the Trinity assembly. Stress value in the lower left-hand corner indicates the disagreement between the plot configuration and the predicted values from the regression. Ellipses represent 95% confidence intervals, with the results of a two-way PERMANOVA (*p* < 0.05) on the dissimilarity matrix of the data presented in the upper right-hand corner, representing the significance of fixed nitrogen and phosphorus availability on relative differences.

**FIGURE 6:**
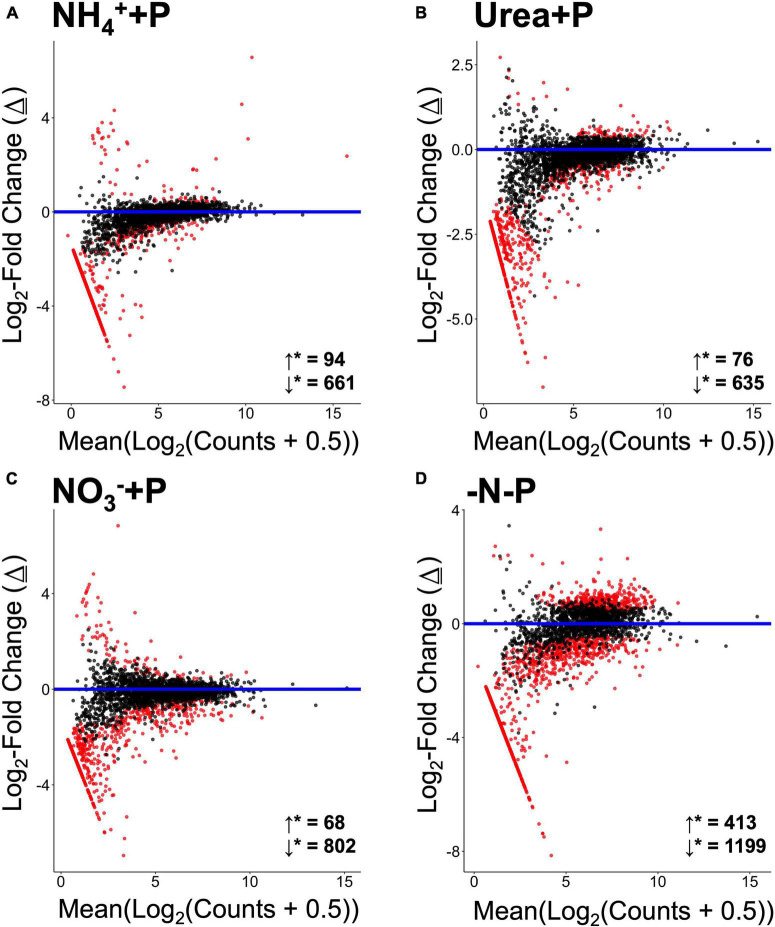
MA plots representing log_2_-fold changes (Δ) in gene expression of experimental treatments [**(A)** = NH_4_^+^ + P, **(B)** = Urea + P, **(C)** = NO_3_^–^ + P, **(D)** = −N-P) relative to the control (−N + P). Change is plotted relative to base mean expression of gene transcripts normalized, log_2_-transformed, annotated and aggregated relative to KO numbers. Significantly differentially upregulated (↓*) genes and downregulated (↓*) genes are indicated directly above the x-axes. Gene transcripts exhibiting statistically significant differences in expression (False Discovery Rate/*qval* < 0.05) are colored red. Statistical analyses were performed in Sleuth using the Wald test.

Non-metric multi-dimensional (nMDS) scaling of normalized read counts mapped to the Trinity assembly revealed considerable overlap among transcriptomes, particularly with respect to NH_4_^+^ + P, which overlapped with urea + P and the control group (-N + P; Figure 5). The transcriptomes of −N-P cultures, in contrast, were significantly different from those of all other treatments (CI = 95%; *p* < 0.05) except those of NH_4_^+^ + P. Furthermore, the transcriptomes of NO_3_^–^ + P were significantly different from those of NH_4_^+^ + P and −N-P cultures (CI = 95%; *p* < 0.05). Fixed N (*p* = 0.001) and P (*p* = 0.05) availability significantly (Two-way PERMANOVA) influenced differences in global gene expression among treatments. All treatments displayed substantially more annotated transcripts that were significantly lower in abundance than transcripts that were significantly higher in abundance relative to the control (Wald test; *qval* < 0.05; Figure 6). The transcriptomes of NH_4_^+^ + P, NO_3_^–^ + P, and urea + P had fewer than 100 transcripts that were significantly more abundant and ∼630 - 810 transcripts that were significantly less abundant (Figures 6A–C). The transcriptomes of −N-P had the largest number of differentially expressed transcripts (Wald test; *qval* < 0.05) with ∼400 and ∼1200 transcripts significantly more and less abundant, respectively (Figure 6D).

### Enriched gene categories and differentially expressed genes

**FIGURE 7:**
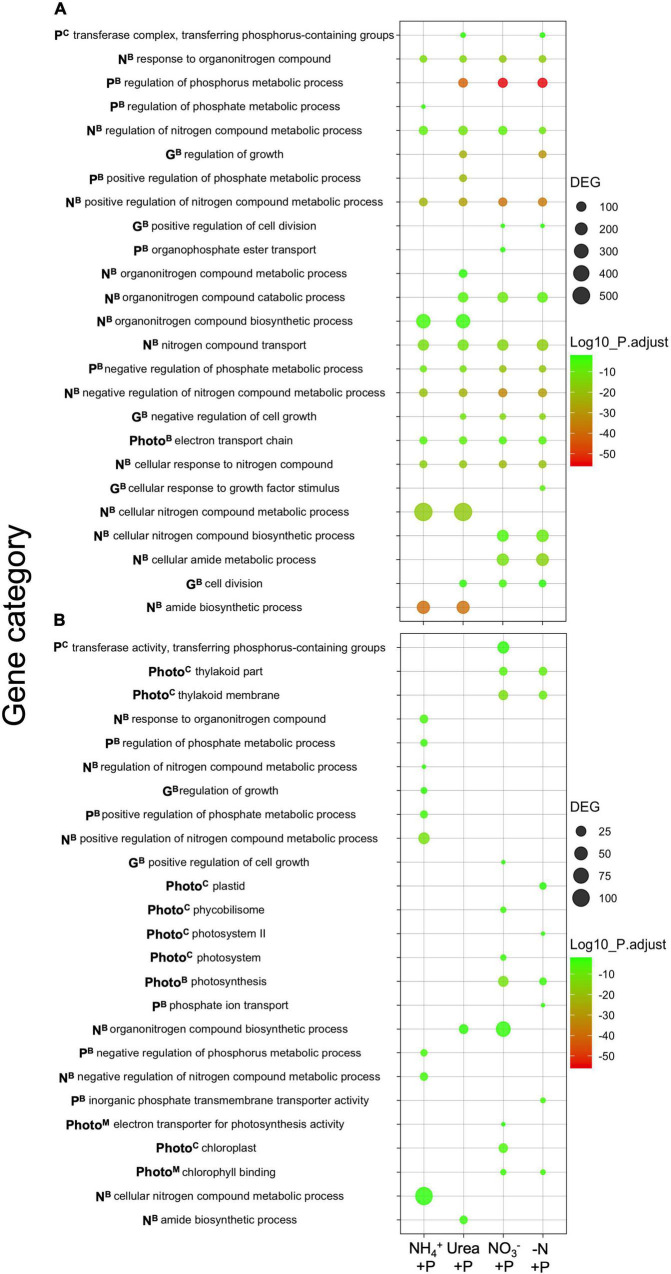
Differentially expressed categories of genes [**(A)** = 4-fold downregulated, **(B)** = 4-fold upregulated] relative to the control (−N + P). Results are from transcript-based Gene Ontology (GO) enrichment analysis. The significant differential expression of genes was determined using Sleuth (Wald test; *qval* < 0.05), and gene categories were tested for significant differential expression using GOenrich (hypergeometric distribution analysis; *padj* < 0.05). *Padj* values were Log_10_-transformed for scaling purposes, and green - red colorations corresponded to lower - higher degrees of significance. Gene categories plotted were those associated with nitrogen (N) and phosphorus (P) assimilation, growth (G), as well as photosynthesis (Photo), while superscripts correspond to biological ^(B)^, cellular ^(C)^, and molecular ^(M)^ gene categories. The number of differentially expressed genes (DEGs) in gene categories are represented by circle size.

Among experimental treatments, the number of significantly downregulated gene categories (hypergeometric distribution analysis; *padj* < 0.05) relative to the control (−N + P) was higher than the number of upregulated gene categories (Figure 7). The transcriptomes of NH_4_^+^ + P exhibited the smallest number of significantly downregulated gene categories associated with fixed N and P transport/metabolism (11; Figure 7A). Transcriptomes in this treatment also had the second highest number of significantly upregulated gene categories (9) after those of NO_3_^–^ + P (11; Figure 7B). Only the transcriptomes of NH_4_^+^ + P and urea + P cultures exhibited significant downregulation of organonitrogen biosynthesis, cellular N metabolism, and amide biosynthesis gene categories; these gene categories exhibited an average number of ∼300, ∼500, ∼200 differentially expressed genes (DEGs), respectively, in both treatments. These genes comprised roughly 18% of the total number (5597) of DEGs that were downregulated across all experimental treatments (Figure 7A). Of these, NH_4_^+^ + P exhibited significant upregulation of 5 out of 7 gene categories associated with N metabolism and biosynthesis, while only 2 and 1 of these gene categories were upregulated in urea + P and NO_3_^–^ + P, respectively, and −N-P exhibited no such enrichment (Figure 7B). NO_3_^–^ + P and −N-P were the only treatments to exhibit significant downregulation of cellular N biosynthesis and cellular amide metabolism gene categories; both gene categories in NO_3_^–^ + P and −N-P exhibited ∼200 DEGs (Figure 7A).

**FIGURE 8:**
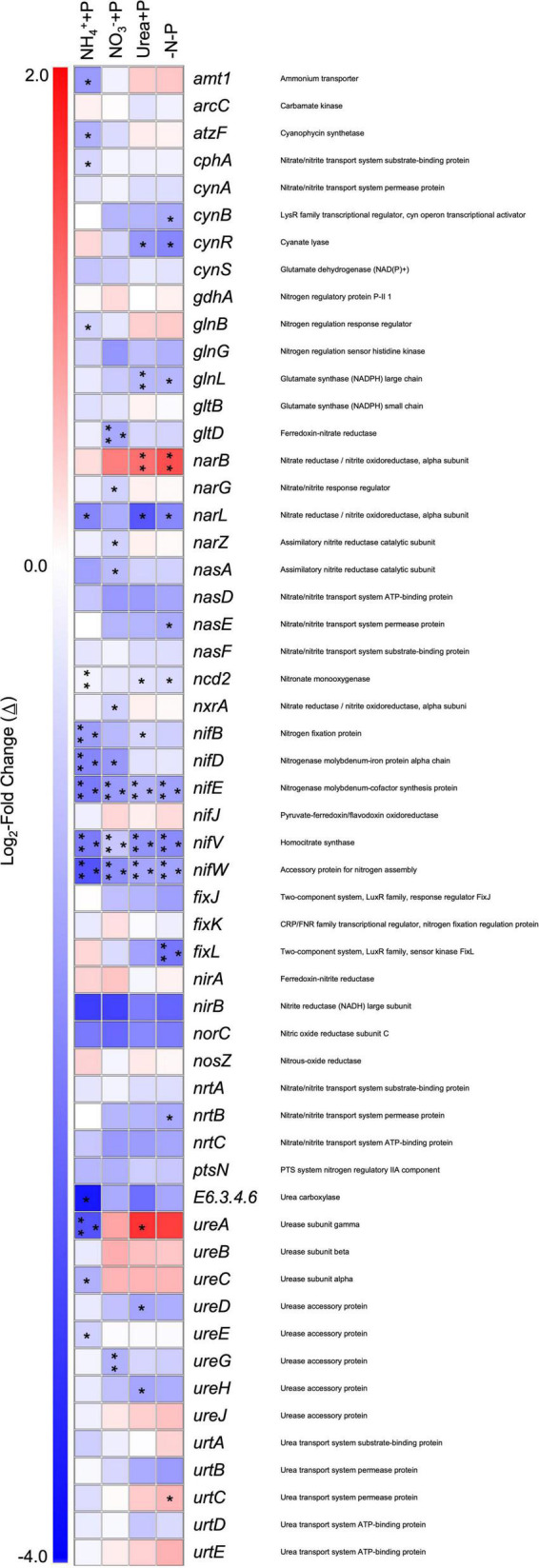
Differential expression of gene transcripts associated with nitrogen metabolism and acquisition in experimental groups relative to the control/−N + P. Heatmap representing log_2_-fold changes (Δ) in nitrogen metabolism/acquisition gene expression in experimental groups with their corresponding gene products. Red or blue coloration corresponds to up or down – regulation of genes relative to the control, respectively. Statistical analyses were determined on transcriptomic data post-Sleuth analysis using the Wald test (*qval* < 0.05 = *, 0.01 = **, 0.001 = ***).

Across all experimental treatments, a majority (> 90%) of annotated transcripts associated with N metabolism were significantly lower (Wald test; *qval* < 0.05) in abundance relative to the control (−N + P; Figure 8). Genes involved in NO_3_^–^ metabolism (*narB*, *narG*, *narL*) were significantly differentially expressed in at least one of the experimental treatments. NO_3_^–^ + P (Δ≅ + 1.11, *qval* < 0.01) and −N-P (Δ≅ + 1.38, *qval* < 0.01) cultures significantly increased the number of transcripts for *narB*, which encodes for ferredoxin−Nitrate reductase. The number of transcripts for *narL*, which encodes for the NO_3_^–^/NO_2_^–^ response regulator, and *narG*, which encodes the Δ-subunit for NO_3_^–^/NO_2_^–^ oxidoreductase, were, respectively, significantly lower in NH_4_^+^ + P (Δ≅−1.89, *qval* < 0.05) and urea + P (Δ≅−0.70 *qval* < 0.05) transcriptomes (Figure 8).

The number of transcripts for *ureA*, which encodes for a subunit of urease, and *urtC*, which encodes for the urea transport system permease, significantly (Wald test; *qval* < 0.05) increased in the transcriptomes of NO_3_^–^ + P (Δ≅ + 1.60) and −N-P (Δ≅ + 0.58), respectively, relative to the control (−N + P; Figure 8). In contrast, *ureA* exhibited a significantly lower number of transcripts in the transcriptomes of NH_4_^+^ + P (Δ≅−2.68, *qval* < 0.001), as did *ureC* (Δ≅−1.23, *qval* < 0.05), and other genes that encode for enzymes associated with urea degradation, namely *atzF* (allophonate hydrolase; Δ≅−1.20, *qval* < 0.05) and *E6.3.4.6* (urea carboxylase; Δ≅−3.62, *qval* < 0.05). Significant differential expression (Δ≅−1.58, *qval* < 0.05) of *amt1*, which encodes for an ammonium transporter, was only observed in the transcriptomes of NH_4_^+^ + P, with transcripts being lower in abundance. Other N metabolism genes that were differentially expressed include *cphA*, which encodes for cyanophycin synthetase, exhibited a significantly lower number of transcripts in the transcriptomes of NH_4_^+^ + P (Δ≅−0.67, *qval* < 0.05), as well as genes that encode for cyanase, *cynB* (Δ≅−1.31, *qval* < 0.05) and *cynR* (Δ≅−1.88, *qval* < 0.05), which exhibited significantly lower transcripts in the transcriptomes of −N-P. The number of transcripts for glutamine synthetase-encoding genes (*gln*) was significantly lower in the transcriptomes of NH_4_^+^ + P (*glnB*; Δ≅−0.70, *qval* < 0.05), NO_3_^–^ + P (*glnL*; Δ≅−1.12, *qval* < 0.01), and −N-P (*glnL*; Δ≅−1.13, *qval* < 0.05), while *gltD* was the only gene involved in glutamate synthesis to exhibit a significantly lower transcript abundance, specifically in the transcriptomes of urea + P (Δ≅−1.35, *qval* < 0.001). Significantly lower numbers of transcripts (Wald test; *qval* < 0.001) of *nifE*, *nifV*, and *nifW*, which encode for nitrogenase subunits, were observed in all experimental treatments (Figure 8). Significantly lower numbers of transcripts of *nifB* (Δ≅−1.52, *qval* < 0.001) and *nifD* (Δ = −1.88, *qval* < 0.001) were observed in NH_4_^+^ + P, while the former and latter genes were present at significantly lower transcript abundances in NO_3_^–^ + P (Δ≅−0.64, *qval* < 0.05) and urea + P (Δ≅−1.62, *qval* < 0.05), respectively, compared to the control. Finally, the transcriptomes of −N-P exhibited significantly lower transcript abundance of *fixL* (Δ≅−2.15, *qval* < 0.001; Figure 8), which encodes for a putative oxygen sensor.

**FIGURE 9:**
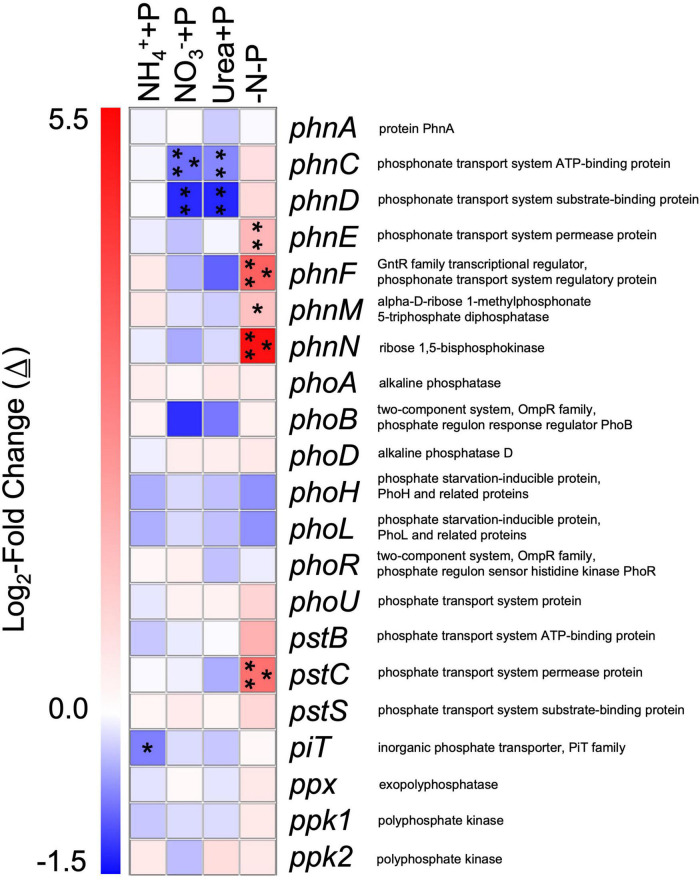
Differential expression of gene transcripts associated with phosphorus acquisition and metabolism in experimental groups relative to the control/−N + P. Heatmap representing log_2_-fold changes (Δ) in phosphorus metabolism/acquisition gene expression in experimental groups with their corresponding gene products. Red or blue coloration corresponds to up or down – regulation of genes relative to the control, respectively. Statistical analyses were determined on transcriptomic data post-Sleuth analysis using the Wald test (*qval* < 0.05 = *, 0.01 = **, 0.001 = ***).

Genes associated with phosphorus (P) assimilation that were differentially expressed relative to the control (−N + P) included those that belong to the *phn*, *pho*, and *pst* clusters (Figure 9). For cultures amended with fixed N, genes that exhibited a significant change (Wald test; *qval* < 0.05) in transcript abundance were always lower than those of the control, while the transcriptomes of −N-P were the only ones to exhibit a significantly greater number of transcripts relative to the control, specifically for four *phn* genes and *pstC* (Figure 9). While non-significant, polyphosphate synthesizing (*ppk1*, Δ≅ + 0.47) and degradation (*ppx*, Δ≅ + 0.49) – encoding genes also exhibited the highest average increase in transcript number in the transcriptomes of −N-P than any other treatment relative to the control (Wald test; *q* > 0.05; Figure 9).

**FIGURE 10:**
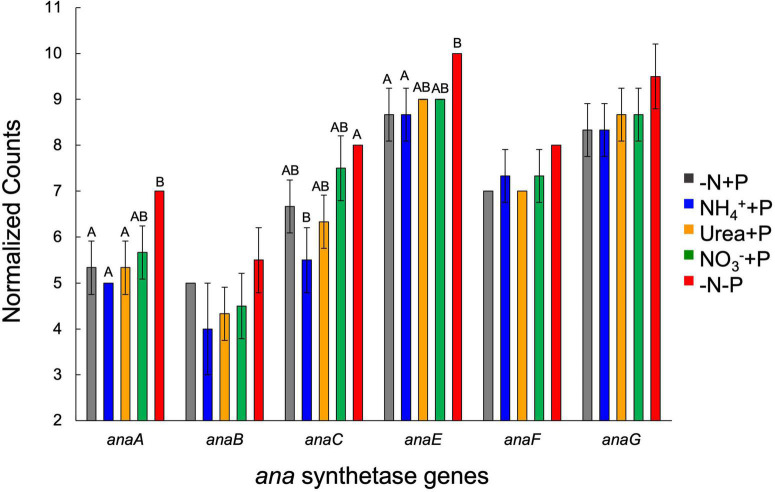
Expression (normalized counts) of genes involved in anatoxin synthesis across treatments at the final timepoint (day 9). Error bars represent standard deviation. Letters above bars represent significant differences between experimental groups (Two-way ANOVA; Tukey’s HSD *post hoc*; *p* < 0.05).

Of the genes associated with ATX-A synthesis, transcript abundances of *anaA*, *anaE*, and *anaF* were significantly (Two-Way ANOVA; *p* < 0.05) altered by P availability, while *anaC* transcript numbers were significantly affected by fixed N and P availability (Figure 10). −N-P cultures exhibited a significantly (Tukey HSD; *p* < 0.05) higher number of *anaA* transcripts (7.0 ± 0.0) relative to those of the control (−N + P; 5.3 ± 0.6) as well as NH_4_^+^ + P (5.0 ± 0.0) and urea + P (5.3 ± 0.6) cultures, while the number of *anaE* transcripts in −N-P cultures (10.0 ± 0.0) was significantly higher than those of the control and NH_4_^+^ + P (8.7 ± 0.6). The number of *anaC* transcripts was highest in −N-P cultures (8.0 ± 0.0) and was significantly higher than that of NH_4_^+^ + P cultures (5.5 ± 0.7, Figure 10).

## Discussion

This study characterized the effects of NH_4_^+^, urea, and NO_3_^–^ as well as P availability on *Dolichospermum* sp. 54. Relative to the control (−N + P), reduced N species significantly increased growth rates and decreased N_2_ fixation rates (NH_4_^+^ + P and urea + P), ATX-A cellular quotas (NH_4_^+^ + P), as well as *nif* (NH_4_^+^ + P and urea + P) transcript abundances. P depletion did not affect growth rate but significantly enhanced ATX-A cellular content and *ana* transcript abundance. We also found a significant negative correlation between ATX-A concentration and growth rate.

### Effects of fixed N and P availability on growth and photosynthetic activity

*Dolichospermum* sp. 54 amended with fixed N exhibited significantly higher maximum growth rates (NO_3_^–^, NH_4_^+^, urea) and *F_*v*_/F_*m*_* (NO_3_^–^ and NH_4_^+^) relative to the control (−N + P) and −N-P cultures. NH_4_^+^ + P and NO_3_^–^ + P were also the only experimental treatments to exhibit significant upregulation of gene categories associated with growth. It has been well established with other *Dolichospermum* (Rhee and Lederman, 1983; Mishra, 1997; Zulkefli and Hwang, 2020) and Nostocales (Presing et al., 1996; Ammar et al., 2014) isolates that NH_4_^+^ and NO_3_^–^ significantly increase growth and decrease N_2_ fixation rates, though NH_4_^+^ is generally considered more effective than NO_3_^–^ in affecting these physiological processes (Stacey et al., 1977; Elder and Parker, 1984). This is not always the case, however, as some members of this order may prefer NO_3_^–^ and urea (Stucken et al., 2014; Qian et al., 2017; Erratt et al., 2018) or even fixed N-deplete conditions (Velzeboer et al., 2001; Molot, 2017) over NH_4_^+^ for growth. The considerable diversity in responses to fixed N among Nostocales stresses the importance of determining how multiple fixed N compounds affect other members of this order, particularly toxin producers such as *Dolichospermum* sp. 54. NO_3_^–^ must be reduced to nitrite (NO_2_^–^) and again to NH_4_^+^ before entering anabolic pathways (Herrero and Flores, 2018). The *F_*v*_/F_*m*_* of NO_3_^–^ + P was not significantly different from that of NH_4_^+^ + P and was the only fixed N-amended treatment to exhibit significant upregulation of photosynthetic gene categories. It is plausible that a significant fraction of the reductants produced by the photosynthetic light reactions in this treatment were consumed by NO_3_^–^ reduction, thus keeping photosynthetic efficiency high, but resulting in a lowered growth rate relative to NH_4_^+^-grown cultures (Flores et al., 1983, 2005).

Urea + P cultures exhibited growth rates that were significantly enhanced relative to the control but not significantly different than those of NH_4_^+^ + P or NO_3_^–^ + P. Strains of CHAB taxa such as *Microcystis* and *Dolichospermum* grown on urea often exhibit enhanced growth rates, toxin production, and/or amino acid biosynthetic rates relative to cells grown with NH_4_^+^ or NO_3_^–^ (Qian et al., 2017; Erratt et al., 2018; Krausfeldt et al., 2019). Aside from two NH_3_ molecules, urea hydrolysis also yields one CO_2_ molecule (Mobley and Hausinger, 1989; Valladares et al., 2002; Veaudor et al., 2019). While urea is potentially both a source of fixed N and carbon for cyanobacterial growth (Krausfeldt et al., 2019), our findings suggest that the increase in *Dolichospermum* sp. 54 growth on this reduced N compound was largely due to the ability to utilize NH_3_ rather than CO_2_.

### Fixed N and P availability effects on N_2_ fixation

While N_2_ fixation can give *Dolichospermum* spp. and other Nostocales taxa a competitive edge over non-diazotrophic cyanobacteria under fixed N-limiting conditions, nitrogenase activity is an energetically demanding process that generally comes at the cost of higher growth rates (Willis et al., 2016; Herrero and Flores, 2018; Wannicke et al., 2021). Traditionally speaking, the productivity of non-diazotrophic phytoplankton was thought to be primarily limited by P (Smith, 1983, 2016; Schindler et al., 2008). Considerable evidence collected over the last decade or so indicates that this is not necessarily the case, as N_2_ fixation alone is insufficient to supply CHAB taxa with N levels sufficient in order to initiate blooms (Scott and McCarthy, 2010; Hellweger et al., 2016; Hayes et al., 2019). While this concept is primarily applied to non-diazotrophic cyanobacteria, our findings indicate that diazotrophs such as *Dolichospermum* exhibit a substantially greater preference for fixed N assimilation over nitrogenase activity, and thus likely play a markedly less important role in supplying newly fixed N to nitrogen-replete freshwater ecosystems during bloom events. The significantly enhanced growth rates of *Dolichospermum* sp. 54 cultured with NO_3_^–^, NH_4_^+^, and urea reported in this study further complements the findings of other studies that have documented Nostocales blooms when fixed N concentrations are high (Chaffin et al., 2019, 2020; Gamez et al., 2019).

N_2_ fixation rates of cultures amended with reduced fixed N (NH_4_^+^ and urea) were significantly lower than those of any other group for the entire experiment, while the N_2_ fixation rates of NO_3_^–^ + P and −N-P were significantly higher than those of the reduced N treatments. These observations, as well as the significant negative correlation between N_2_ fixation rate and growth rate, were consistent with prior studies (Mickelson et al., 1967; Stacey et al., 1977; Ge et al., 1990; Mekonnen et al., 2002). Despite being grown under P-deplete conditions, −N-P cultures exhibited N_2_ fixation rates that were not different from those of the control. The −N-P cultures may have maintained relatively high N_2_ fixation rates over the course of the experiment by hydrolyzing internal P stores (Gerber and Wickstrom, 1990; Burut-Archanai and Powtongsook, 2017), as their transcriptomes exhibited the greatest increase in transcript abundance of polyphosphate synthesis (*ppk1*) and degradation (*ppx*) - encoding genes (Akiyama et al., 1993; Sanz-Luque et al., 2020) of the experimental treatments relative to the control. This, coupled with significantly enhanced APA activity in −N-P relative to control (−N + P) cultures, likely facilitated sustained growth and N_2_ fixation rates that matched the control during the experiment, and further reinforces the widely accepted notion that Nostocales such as *Dolichospermum* have multiple strategies to handle the stresses of P-limitation (Isvanovics et al., 2000; Wu et al., 2012; Lin et al., 2018).

The N_2_ fixation rates of NO_3_^–^ + P cultures were also similar to those of −N treatments when averaged over the entire experiment, strongly indicating that NO_3_^–^ assimilation and nitrogenase activity are utilized simultaneously (Bone, 1971; Elder and Parker, 1984). Supporting this notion, other *Dolichospermum* strains were still reported to exhibit detectable nitrogenase activities when grown at particularly high (1–10 mM) NO_3_^–^ concentrations, whereas those grown at the same or lower concentrations of NH_4_^+^ did not. These studies did not, however, report significantly enhanced growth rates in *Dolichospermum* amended with NH_4_^+^ relative to those grown on NO_3_^–^ (Meeks et al., 1983; Sanz et al., 1995; Mekonnen et al., 2002), while another study reported that NH_4_^+^ derived from NO_3_^–^ reduction, not NO_3_^–^ itself, is ultimately responsible for the suppression of nitrogenase activity (Ramos and Guerrero, 1983). Other studies have reported significantly higher photosynthetic O_2_ evolution (Mishra, 1997) and growth rates (Zulkefli and Hwang, 2020) in NH_4_^+^-grown relative to NO_3_^–^-grown *Dolichospermum*, though in the latter study heterocyst density was significantly higher in NH_4_^+^-grown cultures, suggesting that N_2_ fixation may have been significantly higher. These findings, coupled with differences in growth rate, *F_*v*_/F_*m*_*, and N_2_ fixation rate with respect to fixed N type, reveal how NO_3_^–^, NH_4_^+^, and urea differentially alter physiological processes in *Dolichospermum*.

Differential expression of genes associated with diazotrophy both supported observations regarding N_2_ fixation rates and further provided deeper physiological insight regarding this process. Transcripts of *nifB* and *nifD* were both significantly lower in NH_4_^+^ + P, while the former and latter genes were only significantly downregulated in NO_3_^–^ + P and urea + P, respectively. The gene *nifB* is further associated with Fe-Mo cofactor assemblage (Curatti et al., 2007), while *nifD* encodes for the ɑ-chain for nitrogenase’s Fe-Mo protein (Dos Santos et al., 2012). Significantly lower transcript abundance of *Dolichospermum* sp. 54 *nif* genes in response to NH_4_^+^ amendment and/or PO_4_^–^ depletion is consistent with previous findings in strains of the same genus. While NO_3_^–^ was also reported to have less of an effect on *nif* gene expression than NH_4_^+^ in these findings (Helber et al., 1988; Martin−Níeto et al., 1991), transcriptional changes for multiple genes belonging to this cluster in response to the availability of P and multiple fixed N compounds in other *Dolichospermum* taxa have been poorly characterized. It was also shown that when *Dolichospermum* is grown under fixed N-replete or P-deplete conditions, *nifE* and *nifV*, which encode Fe-Mo cofactor proteins (Hawkes et al., 1984; Ugalde et al., 1984), and accessory protein-encoding *nifW* (Nonaka et al., 2019) are all downregulated. Finally, the significant downregulation of *nifD* in NH_4_^+^P and urea + P cultures, which also exhibited the lowest N_2_ fixation rates among treatments, is likely a reliable indicator of repressed N_2_ fixation when *Dolichospermum* are actively utilizing either N species.

### Effect of fixed N and P availability on ATX-A production

Anatoxin-a (ATX-A) is a highly potent neurotoxin produced by freshwater cyanobacteria with a broad geographic distribution (Devlin et al., 1977; John et al., 2019; Österholm et al., 2020), yet the factors that control the production of this toxin are largely unknown. Several studies suggest that fixed N (Rapala et al., 1993; Gagnon and Pick, 2012) and/or P (Rapala and Sivonen, 1998; Toporowska et al., 2016) availability regulate ATX-A production. There is also evidence to suggest that ATX-A content in cells is controlled by growth rate, which subsequently regulates the partitioning of toxins into daughter cells (Heath et al., 2016), a concept known as the growth differentiation balance hypothesis (Herms and Mattson, 1992). Significant increases (−N-P) and decreases (NH_4_^+^ + P) in the cellular ATX-A content of *Dolichospermum* sp. 54 relative to the control (−N + P) and the significant negative correlation between ATX-A cell quota and growth rate suggests that toxin concentration was driven by division rate. However, expression of the anatoxin synthetase gene (*ana*) cluster indicates that factors other than growth rate regulate anatoxin production. Transcripts of *anaA* as well as *anaC-F* were significantly more abundant under P-deplete conditions, indicating cells were actively increasing toxin synthesis in response to nutrient-limited stress and did not simply accumulate more ATX-A since cells were dividing more slowly. The genes *anaA* and *anaC* encode proteins associated with thioesterase and proline adenylation, respectively, while *anaE* and *anaF* both encode for type I polyketide synthases (Rantala-Ylinen et al., 2011). Of the four genes, *anaC* best predicted variance in ATX-A cellular content among treatments, as both toxin quota and transcript abundance of *anaC* were significantly lower due to NH_4_^+^ enrichment and increased in response to P depletion. These data, when considered with reports of *anaC* expression exhibiting a significant negative relationship with dissolved carbon-to−Nitrogen (C:N) ratios (Tao et al., 2020), suggests that *anaC* expression and subsequent ATX-A production is highly sensitive to fixed N availability. Ecologically speaking, such an active process could serve as an defense mechanism against grazers (Toporowska et al., 2014; Anderson et al., 2018) or an allelopathic strategy against other phytoplankton (Kearns and Hunter, 2001; Chia et al., 2018, 2019), particularly if anatoxin-producing cyanobacteria are subjected to conditions unsuitable for them to achieve optimal growth rates (Harland et al., 2013; Heath et al., 2016).

The toxin content of *Dolichospermum* sp. 54 also exhibited a positive correlation (*p* < 0.06) with nitrogenase activity, which was driven by experimental treatments with lower growth rates exhibiting higher N_2_ fixation rates and cellular toxin content. The manner in which anatoxin synthetase may interact with other biochemical pathways is poorly understood (Rantala-Ylinen et al., 2011). However, given that different N forms alter growth, photosynthesis, and toxin production in *Dolichospermum* sp. 54 and other cyanobacteria (Harke et al., 2016; Erratt et al., 2018), understanding how nitrogenase activity might directly influence anatoxin production is warranted. To our knowledge, no study has investigated whether nitrogenase activity influences ATX-A synthesis. It is also important to note that other Nostocales taxa have exhibited significantly higher ATX-A concentrations and growth rates when grown on urea rather than NH_4_^+^ and NO_3_^–^ (Qian et al., 2017; Tao et al., 2020). Urea + P cultures exhibited no significant difference in ATX-A cell quota relative to the control. This suggests that the ability for cyanobacteria to increase anatoxin production when using urea as a fixed N source may be dependent on whether they can fix CO_2_ derived from urease activity (Krausfeldt et al., 2019), as ATX-A is a toxin with a high C:N ratio (Van de Waal et al., 2014). Tao et al. (2020), who observed significantly higher growth rates and ATX-A quotas in the Nostocales taxon *Cuspidothrix issatschenkoi* when cultures were grown on urea relative to other fixed N sources, suggested as much in their discussion. Consistent with our findings, they further reported that *F_*v*_/F_*m*_* was significantly lower in urea-grown cultures than those grown on NO_3_^–^ (Tao et al., 2020), which further reinforces the notion that reductants from photosynthetic light reactions can be used to reduce NO_3_^–^ (Flores et al., 1983, 2005) but not convert urea to NH_4_^+^.

### Global transcriptomic changes in response to fixed N and P availability

The transcriptomes of fixed N-amended cultures exhibited considerable similarity to each other and to the control, while the transcriptomes of −N-P were the most dissimilar from other treatments and exhibited the greatest number of differentially expressed genes relative to the control. In addition, all experimental treatments exhibited a greater number of downregulated genes than upregulated genes relative to −N + P transcriptomes. These findings complement those of previous studies, in which removal of NO_3_^–^ or NH_4_^+^ in *Dolichospermum* cultures led to a greater number of significantly upregulated rather than downregulated genes associated with heterocyst production as well as stress responses to fixed N deprivation (Ehira and Ohmori, 2006; Flaherty et al., 2011; Mitschke et al., 2011). However, it is important to note that the −N-P treatment was the least different from the control relative to growth, *F_*v*_/F_*m*_*, N_2_ fixation, and ATX-A quota. Thus, it is likely that fixed N and P deprivation significantly influenced components of the *Dolichospermum* transcriptome beyond genes associated with growth, toxin production, and nitrogen assimilation.

### Effects of nutrient availability on N and P assimilation

N form as well as P depletion led to significant differential expression of specific genes associated with N metabolism and N_2_ fixation relative to the control. The downregulation of *amt1*, which encodes for an ammonium transporter, and *cphA*, which encodes for cyanophycin synthetase, in NH_4_^+^ + P relative to the control, for instance, is consistent with previous findings, as cyanobacteria generally upregulate these genes only when fixed N is scarce (Montesinos et al., 1998; Muro-Pastor et al., 2005; Paz-Yepes et al., 2008). Nutrient availability also significantly affected the expression of genes associated with the glutamine synthetase-glutamate synthase (GS-GOGAT) pathway, a major metabolic process by which NH_4_^+^ is assimilated (Wolk et al., 1976). For example, *glnB*, which encodes for the regulatory protein PII (Forchhammer, 2004), was downregulated in the transcriptomes of NH_4_^+^ + P, which is consistent with previous findings (Tsinoremas et al., 1991; Paz-Yepes et al., 2009). PII activity is essential to the C:N balance in cyanobacteria and increases with increasing levels of 2-oxoglutarate, which is an indicator of the balance between CO_2_ fixation and NH_4_^+^ assimilation (Muro-Pastor et al., 2001; Forchhammer, 2004; Valladares et al., 2008). 2-oxoglutarate levels also regulate the activity of NtcA, a global transcriptional regulator for N assimilation pathways (Zhao et al., 2010; Picossi et al., 2014). The activity and levels of NtcA and 2-oxoglutarate, respectively, were not measured in this study. However, *gltD*, which encodes for glutamate synthase (Goss et al., 2001) was significantly downregulated in urea + P relative to the control. As glutamate is produced by glutamate synthase transferring nitrogen from glutamine to 2-oxoglutarate (Walker and van der Donk, 2016), it is highly likely that 2-oxoglutarate levels, as well as NtcA activity, are significantly affected when P-replete *Dolichospermum* sp. 54 are given urea as a fixed N source.

Downregulation of *glnL*, which encodes for nitrogen regulator II (NRII) (Ninfa and Magasanik, 1986), was observed in the transcriptomes of NO_3_^–^ + P and −N-P. Downregulation of *gltD*, which encodes for part of the NADPH-GOGAT (Okuhara et al., 1999), was also observed in the transcriptomes of urea + P. The significant downregulation of *glnL* expression suggests that glutamine synthesis significantly decreased in these treatments (Reitzer and Magasanik, 1985; Ninfa and Magasanik, 1986), and reinforces the notion that a large portion of N derived from NO_3_^–^/NO_2_^–^ reduction is not incorporated into organic compounds but rather utilized to promote PSII efficiency as oxidants (Flores et al., 2005). This is likely due to *Dolichospermum* requiring considerable amounts of energy to completely reduce NO_3_^–^ (Ramos and Guerrero, 1983; Elder and Parker, 1984) and accounts for the significantly slower growth rates of NO_3_^–^-grown cells relative to NH_4_^+^-grown cells. Differential expression of genes within the GS-GOGAT pathway in different N treatments provides insight regarding how each fixed N compound affected N and C metabolism in *Dolichospermum* sp. 54.

Genes that encode for nitrate (*narB*) and nitrite (*nirA*) reductases (Cai and Wolk, 1997; Herrero and Flores, 2018) also exhibited significant upregulation and no significant difference in expression, respectively, in NO_3_^–^ + P and −N-P cultures. This suggests that while NO_3_^–^ was being reduced to NO_2_^–^ (*narB*), NO_2_^–^ reduction to NH_4_^+^ (*nirA*) was occurring at a relatively lower rate, which likely reflects NO_3_^–^ + P cultures growing at a significantly slower rate than NH_4_^+^ + P cultures. Regardless, upregulation of *narB* likely reflects the significantly higher *F_*v*_/F_*m*_* and the significant enrichment of gene categories associated with the photosynthetic apparatus in NO_3_^–^ + P cultures than that of the control, as NO_3_^–^ reduction enhances cyanobacterial PSII efficiency as a Hill reagent (Serrano et al., 1981; Flores et al., 1983, 2005). While enhanced nitrate reductase activity in another *Dolichospermum* strain was reported to be essential in inhibiting nitrogenase activity (Martin−Níeto et al., 1991), that was not the case for NO_3_^–^ + P cultures of *Dolichospermum* sp. 54, which exhibited significantly higher transcript abundance of *narB* and no significant change in N_2_ fixation relative to the control. This suggests that other factors, such as the full reduction of NO_3_^–^ to NH_4_^+^, are necessary to inhibit nitrogenase activity in members of this genus to a degree comparable to *Dolichospermum* amended with NH_4_^+^ and urea.

Gene categories associated with PO_4_^3–^ transport were significantly upregulated in the transcriptomes of −N-P relative to the control, a response that is consistent with the effect of P-depletion on other Nostocales taxa (Teikari et al., 2015; Dong et al., 2019). The −N-P treatment, which exhibited significantly higher APA activity relative to the control, was the only treatment to exhibit significant upregulation of several *phn* genes and *pstC*. The *phn* gene cluster encodes for proteins involved in the transport and metabolism of phosphonates under P-deplete conditions and/or components of the carbon-phosphorus lyase, while *pstC* encodes for a high affinity PO_4_^3–^ transporter (Adams et al., 2008; Voß et al., 2013). Collectively, differentially expressed genes associated with P transport and assimilation are consistent with prior studies of gene markers of P-limitation in cyanobacteria (Harke et al., 2012; Harke and Gobler, 2013; Lu et al., 2019) and suggests they are important in supporting *Dolichospermum* blooms when dissolved inorganic phosphorus is scarce.

## Conclusion

In summary, fixed N-replete *Dolichospermum* sp. 54 cultures exhibited significantly enhanced growth (NH_4_^+^, urea, NO_3_^–^) and photosynthetic efficiency (NH_4_^+^ and NO_3_^–^) relative to those grown under fixed N-limiting conditions. NH_4_^+^-grown cultures also exhibited significantly higher and lower growth and N_2_ fixation rates, respectively, relative to NO_3_^–^-grown cultures. Cultures also exhibited significant differences in growth, *F_*v*_/F_*m*_*, N_2_ fixation, ATX-A production, and genome expression depending on whether they were amended with NH_4_^+^, urea, NO_3_^–^, or deprived of both fixed N and P, reinforcing the notion that *Dolichospermum* and likely other toxin-producing Nostocales taxa exhibit highly specific preferences for different fixed N species. These findings also support the growing consensus that diazotrophic cyanobacteria exhibit fixed N-limitation similar to non-diazotrophic CHAB taxa such as *Microcystis* with respect to growth rate, as well as P-limitation, and prefer certain fixed N species under P-replete conditions. From a managerial perspective, if fixed N inputs are not controlled in eutrophic systems where *Dolichospermum* spp. occur, blooms of these and other Nostocales taxa may be of greater biomass and subsequently have higher toxin concentrations.

## Data availability statement

The datasets presented in this study can be found in online repositories. The names of the repository/repositories and accession number(s) can be found in the article/Supplementary material.

## Author contributions

BK and JJ performed the culture experiment and analyzed growth rate, ATX-A, N_2_ fixation, nutrient, and *F_*v*_/F_*m*_* data. BK graphed the data and drafted the manuscript. JJ extracted RNA. MH and DN processed transcriptomic data post-sequencing. CG provided the funding and technical resources necessary to perform experiments and performed the analyses. All authors contributed to data analysis and study design.

## Footnotes

1
https://transdecoder.github.io/

2
https://vicbioinformatics.com/software.prokka.shtml

3
https://github.com/vegandevs/vegan

4
https://software.broadinstitute.org/morpheus/

## Supplementary material

Supplementary Figure 1 Cell densities for all treatments during the experiment, from day 1 to the final time point (day 9). Error bars represent standard deviation.

Supplementary Figure 2 Photosystem II photosynthetic efficiency (*F_*v*_/F_*m*_*) for all treatments during the experiment. Error bars represent standard deviation. Red arrows represent days when fixed N species (NH_4_Cl, Urea, and NaNO_3_) were added to fixed N-replete treatments.

Supplementary Figure 3 Alkaline phosphatase activity (nmol mL^–1^ hr^–1^) as a function of volume and time in treatments deprived on nitrogen and either with or without phosphorus on days 1, 3, 5, and 9. Error bars represent standard error. Number of asterisks correspond to degree of significance (*p* < 0.05 = *, 0.01 = **, 0.001 = ***), determined via Student’s paired *t*-test.

## References

[B1] Adams M. Gomez-Garcia M. Grossman A. Bhaya D. (2008). Phosphorus deprivation responses and phosphonate utilization in a thermophilic *synechococcus* sp. from microbial mats. *J. Bacteriol.* 190 8171–8184. 10.1128/JB.01011-08 18931115 PMC2593230

[B2] Akiyama M. Crooke E. Kornberg A. (1993). An exopolyphosphatase of *Escherichia coli*. *J. Biol. Chem.* 268 633–639. 10.1016/S0021-9258(18)54198-3 8380170

[B3] Ammar M. Comte K. Tran T. El Bour M. (2014). Initial growth phases of two bloom-forming cyanobacteria (*Cylindrospermopsis raciborskii* and *Planktothrix agardhii*) in monocultures and mixed cultures depending on light and nutrient conditions. *Ann. Limnol. Int. J. Lim.* 50 231–240. 10.1051/LIMN/2014096

[B4] Anderson B. Voorhees J. Phillips B. Fadness R. Stancheva R. Nichols J. (2018). Extracts from benthic anatoxin-producing *Phormidium* Are Toxic to 3 macroinvertebrate taxa at environmentally relevant concentrations. *Environ. Toxicol. Chem.* 37 2851–2859. 10.1002/etc.4243 30066467

[B5] Bankevich A. Nurk S. Antipov D. Gurevich A. Dvorkin M. Kulikov A. (2013). SPAdes: A new genome assembly algorithm and its applications to single-cell sequencing. *J. Comput. Biol.* 19 455–477. 10.1089/cmb.2012.0021 22506599 PMC3342519

[B6] Beversdorf L. Miller T. McMahon K. (2013). The role of nitrogen fixation in cyanobacterial bloom toxicity in a temperate, eutrophic lake. *PLoS One* 8:e56103. 10.1371/journal.pone.0056103 23405255 PMC3566065

[B7] Blomqvist S. Gunnars A. Elmgren R. (2004). Why the limiting nutrient differs between temperate coastal seas and freshwater lakes: A matter of salt. *Limnol. Oceanogr.* 49 2236–2241. 10.4319/lo.2004.49.6.2236

[B8] Bolger A. Lohse M. Usadel B. (2014). Trimmomatic: A flexible trimmer for Illumina sequence data. *Bioinformatics* 30 2114–2120. 10.1093/bioinformatics/btu170 24695404 PMC4103590

[B9] Bone D. (1971). Nitrogenase activity and nitrogen assimilation in *anabaena flos-aquae* growing in continuous culture. *Arch. Microbiol.* 80 234–241. 10.1007/BF00410124 5002351

[B10] Bray N. Pimentel H. Melsted P. Pachter L. (2016). Near-optimal probablistic RNA-seq quantification. *Nat. Biotechnol.* 34 525–527. 10.1038/nbt.3519 27043002

[B11] Buckland P. Codd G. Hall T. Izydorczyk K. Kull T. Lindholm T. (2005). *Toxic: Cyanobacterial monitoring and cyanotoxin analysis.* Åbo: Åbo Akademi University Press.

[B12] Burut-Archanai S. Powtongsook S. (2017). Identification of negative regulator for phosphate-sensing system in *Anabaena* sp. PCC 7120: A target gene for developing phosphorus removal. *Biochem. Eng. J.* 125 129–134. 10.1016/j.bej.2017.05.019

[B13] Bushmanova E. Antipov D. Lapidus A. Prjibelski A. (2019). rnaSPAdes: A de novo transcriptome assembler and its application to RNA-Seq data. *GigaScience* 8:giz100. 10.1093/gigascience/giz100 31494669 PMC6736328

[B14] Cai Y. Wolk C. (1997). Nitrogen deprivation of *Anabaena* sp. strain PCC 7120 elicits rapid activation of a gene cluster that is essential for uptake and utilization of nitrate. *J. Bacteriol.* 179 258–266. 10.1128/jb.179.1.258-266.1997 8982006 PMC178687

[B15] Capone D. (1993). “Determination of nitrogenase activity in aquatic samples using the acetylene reduction procedure,” in *Handbook in methods in aquatic microbial ecology*, eds Kemp P. Cole J. Sherr B. Sherr E. (Boca Raton, FL: CRC Press), 621–631.

[B16] Carmichael W. (1994). The toxins of cyanobacteria. *Sci. Am.* 270 64–70. 10.1038/scientificamerican0194-78 8284661

[B17] Carmichael W. Briggs D. Peterson M. (1979). Pharmacology of anatoxin-a, produced by the freshwater cyanophyte *Anabaena flos-aquae* NRC-44-1. *Toxicon* 17 229–236. 10.1016/0041-0101(79)90212-5 112722

[B18] Chaffin J. Mishra S. Kane D. Bade D. Stanislawczyk K. Slodysko K. (2019). Cyanobacterial blooms in the central basin of lake Erie: Potentials for cyanotoxins and environmental drivers. *J. Great Lakes Res.* 45 277–289. 10.1016/j.jglr.2018.12.006

[B19] Chaffin J. Stanislawczyk K. Kane D. Lambrix M. (2020). Nutrient addition effects on chlorophyll a, phytoplankton biomass, and heterocyte formation in Lake Erie’s central basin during 2014–2017: Insights into diazotrophic blooms in high nitrogen water. *Freshw. Biol.* 65 2154–2168. 10.1111/fwb.13610

[B20] Chia M. Jankowiak J. Kramer B. Goleski J. Huang I. Zimba P. (2018). Succession and toxicity of *Microcystis* and *Anabaena* (*Dolichospermum*) blooms are controlled by nutrient-dependent allelopathic interactions. *Harmful Algae* 74 67–77. 10.1016/j.hal.2018.03.002 29724344

[B21] Chia M. Kramer B. Jankowiak J. Bittencourt-Oliveira M. Gobler C. (2019). The individual and combined effects of the cyanotoxins, anatoxin-A and microcystin-LR, on the growth, toxin production, and nitrogen fixation of prokaryotic and eukaryotic algae. *Toxins* 11:43. 10.3390/toxins11010043 30650515 PMC6357180

[B22] Christensen V. Kahn E. (2020). Freshwater neurotoxins and concerns for human, animal, and ecosystem health: A review of anatoxin-a and saxitoxin. *Sci. Total Environ.* 736:139515. 10.1016/j.scitotenv.2020.139515 32485372

[B23] Correll D. (1999). Phosphorus: A rate limiting nutrient in surface waters. *Poult. Sci.* 78 674–682. 10.1093/ps/78.5.674 10228963

[B24] Curatti L. Hernandez J. Igarashi R. Soboh B. Zhao D. Rubio L. (2007). In vitro synthesis of the iron molybdenum cofactor of nitrogenase from iron, sulfur, molybdenum, and homocitrate using purified proteins. *Proc. Natl. Acad. Sci. U.S.A.* 104, 17626–17631. 10.1073/pnas.0703050104 17978192 PMC2077076

[B25] Dai G. Deblois C. Liu S. Juneau P. Qiu B. (2008). Differential sensitivity of five cyanobacterial strains to ammonium toxicity and its inhibitory mechanism on the photosynthesis of rice-field cyanobacterium Ge-Xian-Mi (Nostoc). *Aquat. Toxicol.* 89, 113–121. 10.1016/j.aquatox.2008.06.007 18640729

[B26] Dell’Aversano C. Hess P. Quilliam M. (2005). Hydrophilic interaction liquid chromatography–mass spectrometry for the analysis of paralytic shellfish poisoning (PSP) toxins. *J. Chromatogr. A.* 1081 190–201. 10.1016/j.chroma.2005.05.056 16038209

[B27] Dempster E. Pryor K. Francis D. Young J. Rogers H. (1999). Rapid DNA extraction from ferns for PCR-based analyses. *Biotechniques* 27 66–68. 10.2144/99271bm13 10407666

[B28] Devlin J. Edwards O. Gorham P. Hunter N. Pike R. Stavric B. (1977). Anatoxin-a, a toxic alkaloid from *Anabaena flos-aquae* NRC-44h. *Can. J. Chem.* 55 1367–1371. 10.1139/v77-189

[B29] Dolman A. M. Rucker J. Pick F. R. Fastner J. Rohrlack T. Mischke U. (2012). Cyanobacteria and cyanotoxins: The influence of nitrogen versus phosphorus. *PLoS One* 7:e38757. 10.1371/journal.pone.0038757 22719937 PMC3376147

[B30] Dong C. Zhang H. Yang Y. He X. Liu L. Fu J. (2019). Physiological and transcriptomic analyses to determine the responses to phosphorus utilization in *Nostoc* sp. *Harmful Algae* 84 10–18. 10.1016/j.hal.2019.03.002 31128794

[B31] Dos Santos P. Fang Z. Mason S. Setubal J. Dixon R. (2012). Distribution of nitrogen fixation and nitrogenase-like sequences amongst microbial genomes. *BMC Genom.* 13:162. 10.1186/1471-2164-13-162 22554235 PMC3464626

[B32] Dyhrman S. Ammerman J. Van Mooy B. (2007). Microbes and the Marine Phosphorus Cycle. *Oceanography* 20 110–116. 10.5670/oceanog.2007.54

[B33] Ehira S. Ohmori M. (2006). NrrA, a nitrogen-responsive response regulator facilitates heterocyst development in the cyanobacterium *Anabaena* sp. strain PCC 7120. *Mol. Microbiol.* 59 1692–1703. 10.1111/j.1365-2958.2006.05049.x 16553876

[B34] Ehira S. Ohmori M. Sato N. (2003). Genome-wide expression analysis of the responses to nitrogen deprivation in the heterocyst-forming cyanobacterium *Anabaena* sp. strain PCC 7120. *DNA Res.* 10 97–113. 10.1093/dnares/10.3.97 12886952

[B35] Elder R. Parker M. (1984). Growth Response of a Nitrogen Fixer (*Anabaena flos-aquae*, Cyanophygeae) to Low Nitrate. *J. Phycol.* 20 296–301. 10.1111/j.0022-3646.1984.00296.x

[B36] El-Shehawy R. Kleiner D. (2003). The mystique of irreversibility in cyanobacterial heterocyst formation:parallels to differentiation and senescence in eukaryotic cells. *Physiol. Plant* 119 49–55. 10.1128/9781555818166.CH3

[B37] Erratt K. Creed I. Trick C. (2018). Comparative effects of ammonium, nitrate and urea on growth and photosynthetic efficiency of three bloom-forming cyanobacteria. *Freshw. Biol.* 63 626–638. 10.1111/fwb.13099

[B38] Falcon S. Gentleman R. (2007). Using GOstats to test gene lists for GO term association. *Bioinformatics* 23 257–258. 10.1093/bioinformatics/btl567 17098774

[B39] Flaherty B. Nieuwerburgh F. Head S. Golden J. (2011). Directional RNA deep sequencing sheds new light on the transcriptional response of *Anabaena* sp. strain PCC 7120 to combined−Nitrogen deprivation. *BMC Genom.* 12:332. 10.1186/1471-2164-12-332 21711558 PMC3141674

[B40] Flores E. Frias J. Rubio L. Herrero A. (2005). Photosynthetic nitrate assimilation in cyanobacteria. *Photosynth. Res.* 83 117–133. 10.1007/s11120-004-5830-9 16143847

[B41] Flores E. Guerrero M. Losada M. (1983). Photosynthetic nature of nitrate uptake and reduction in the cyanobacterium Anacystis nidulans. *BBA* 722 408–416. 10.1016/0005-2728(83)90056-7 19007922

[B42] Fogg G. (1942). Studies on nitrogen fixation by blue-green Algae I. nitrogen fixation by anabaena cylindrica LEMM. *J. Exp. Biol.* 19 78–87. 10.1242/jeb.19.1.78

[B43] Forchhammer K. (2004). Global carbon/nitrogen control by PII signal transductionin cyanobacteria: From signals to targets. *FEMS Microbiol. Rev.* 28 319–333. 10.1016/j.femsre.2003.11.001 15449606

[B44] Gagnon A. Pick F. (2012). Effect of nitrogen on cellular production and release of the neurotoxin anatoxin-a in a nitrogen-fixing cyanobacterium. *Front. Microbiol.* 3:211. 10.3389/fmicb.2012.00211 22701451 PMC3373148

[B45] Gamez T. Benton L. Manning S. (2019). Observations of two reservoirs during a drought in central Texas, USA: Strategies for detecting harmful algal blooms. *Ecol. Indic.* 104 588–593. 10.1016/j.ecolind.2019.05.022

[B46] Ge X. Cain K. Hirschberg R. (1990). Urea metabolism and urease regulation in the cyanobacterium *Anabaena variabilis*. *Can. J. Microbiol.* 36 218–222. 10.1139/m90-037

[B47] Gerber B. Wickstrom C. (1990). Response of *Anabaena flos-aquae* (Cyanophyta) nitrogenase activity to sudden phosphate deprivation. *J. Phycol.* 26 650–655. 10.1111/j.0022-3646.1990.00650.x

[B48] Glibert P. Maranger R. Sobota D. Bouwan L. (2014). The haber bosch-harmful algal bloom (HB-HAB) link. *Environ. Res. Lett.* 9:105001. 10.1088/1748-9326/9/10/105001

[B49] Gobler C. J. Burkholder J. M. Davis T. W. Harke M. J. Johengen T. Stow C. A. (2016). The dual role of nitrogen supply in controlling the growth and toxicity of cyanobacterial blooms. *Harmful Algae* 54 87–97. 10.1016/j.hal.2016.01.010 28073483

[B50] Goss T. Perez-Matos A. Bender R. (2001). Roles of Glutamate Synthase, gltBD, and gltF in Nitrogen Metabolism of *Escherichia coli* and *Klebsiella aerogenes*. *J. Bacteriol.* 183 6607–6619. 10.1128/JB.183.22.6607-6619.2001 11673431 PMC95492

[B51] Grabherr M. Haas B. Yassour M. Levin J. Thompson D. Amit I. (2011). Trinity: Reconstructing a full-length transcriptome without a genome from RNA-Seq data. *Nat. Biotechnol.* 29 644–652. 10.1038/nbt.1883 21572440 PMC3571712

[B52] Guillard R. (1973). “Division Rates,” in *Handbook of phycological methods*, ed. Stein J. (Cambridge: Cambridge Universty Press), 289–312.

[B53] Haas B. Papanicolaou A. Yassour M. Grabherr M. Blood P. Bowden J. (2013). De novo transcript sequence reconstruction from RNA-seq using the Trinity platform for reference generation and analysis. *Nat. Protoc.* 8 1494–1512. 10.1038/nprot.2013.084 23845962 PMC3875132

[B54] Hardy R. Burns R. Holsten R. (1973). Applications of the acetylene-ethylene assay for measurement of nitrogen-fixation. *Soil. Biol. Biochem.* 5 47–81. 10.1104/pp.43.8.1185 16656902 PMC1086994

[B55] Harke M. Berry D. Ammerman J. Gobler C. (2012). Molecular response of the bloom-forming cyanobacterium, *Microcystis aeruginosa*, to phosphorus limitation. *Microb. Ecol.* 63 188–198. 10.1007/s00248-011-9894-8 21720829

[B56] Harke M. Gobler C. (2013). Global transcriptional responses of the toxic cyanobacterium, *Microcystis aeruginosa*, to nitrogen stress, phosphorus stress, and growth on organic matter. *PLoS One* 8:e69834. 10.1371/journal.pone.0069834 23894552 PMC3720943

[B57] Harke M. J. Davis T. W. Watson S. B. Gobler C. J. (2016). Nutrient-controlled niche differentiation of Western Lake Erie cyanobacterial populations revealed via metatranscriptomic surveys. *Environ. Sci. Technol.* 50 604–615. 10.1021/acs.est.5b03931 26654276

[B58] Harland F. Wood S. Moltchanova E. Williamson W. Gaw S. (2013). *Phormidium autumnale* growth and anatoxin-a production under iron and copper stress. *Toxins* 5 2504–2521. 10.3390/toxins5122504 24351714 PMC3873698

[B59] Hawkes T. McLean P. Smith B. (1984). Nitrogenase from nifV mutants of *Klebsiella pneumoniae* contains an altered form of the iron-molybdenum cofactor. *Biochem. J.* 217 317–321. 10.1042/bj2170317 6320803 PMC1153212

[B60] Hayes N. Patoine A. Haig H. Simpson G. Swarbrick V. Wiik E. (2019). Spatial and temporal variation in nitrogen fixation and its importance to phytoplankton in phosphorus-rich lakes. *Freshw. Biol.* 64 269–283. 10.1111/fwb.13214

[B61] He H. Miao R. Huang L. Jiang H. Cheng Y. (2021). Vegetative cells may perform nitrogen fixation function under nitrogen deprivation in *Anabaena* sp. strain PCC 7120 based on genome-wide differential expression analysis. *PLoS One* 16:e0248155. 10.1371/journal.pone.0248155 33662009 PMC7932525

[B62] Heath M. Wood S. Young R. Ryan K. (2016). The role of nitrogen and phosphorus in regulating *Phormidium* sp. (cyanobacteria) growth and anatoxin production. *FEMS Microbiol. Ecol.* 92:fiw021. 10.1093/femsec/fiw021 26862139

[B63] Helber J. Johnson T. Yarbrough L. Hirschberg R. (1988). Effect of nitrogenous compounds on nitrogenase gene expression in anaerobic cultures of *Anabaena variabilis*. *J. Bacteriol.* 170 558–563. 10.1128/jb.170.2.558-563.1988 3123457 PMC210690

[B64] Hellweger F. Fredrick N. McCarthy M. Gardner W. Wilhelm S. Paerl H. (2016). Dynamic, mechanistic, molecular-level modelling of cyanobacteria: *Anabaena* and nitrogen interaction. *Environ. Microbiol.* 18 2721–2731. 10.1111/1462-2920.13299 27059435

[B65] Herms D. Mattson W. (1992). The Dilemma of Plants: To Grow or Defend. *Q. Rev. Biol.* 67 283–335. 10.1086/417659

[B66] Herrero A. Flores E. (2018). Genetic responses to carbon and nitrogen availability in *Anabaena*. *Environ. Microbiol.* 21 1–17. 10.1111/1462-2920.14370 30066380

[B67] Hoffman B. Lukoyanov D. Yang Z. Dean D. Seefeldt L. (2014). Mechanism of nitrogen fixation by nitrogenase: The next stage. *Chem. Rev.* 114 4041–4062. 10.1021/cr400641x 24467365 PMC4012840

[B68] Hoppe H. (1983). Significance of exoenzymatic activities in the ecology of brackish water: Measurements by means of methylumbelliferyl-substrates. *Mar. Ecol. Prog. Ser.* 11 299–308. 10.3354/meps011299

[B69] Howarth R. Marino R. Lane J. Cole J. (1988). Nitrogen fixation in freshwater, estuarine, and marine ecosystems. 1. Rates and importance. *Limnol. Oceanogr.* 33 669–687. 10.4319/lo.1988.33.4part2.0669

[B70] Humpage A. Rositano J. Bretag A. Brown R. Baker P. Nicholson B. (1994). Paralytic shellfish poisons from Australian cyanobacterial blooms. *Mar. Freshwater Res.* 45 761–771. 10.1071/MF9940761

[B71] Hyman M. Arp D. (1987). Quantification and removal of some contaminating gases from acetylene used to study gas-utilizing enzymes and microorganisms. *Appl. Environ. Microbiol.* 53 298–303. 10.1128/aem.53.2.298-303.1987 16347278 PMC203655

[B72] Issa A. Abd-Alla M. Ohyama T. (2014). “Nitrogen fixing cyanobacteria: Future prospect,” in *Advances in biology and ecology of nitrogen fixation*, ed. Takuji O. (London: InTechOpen), 23–48. 10.1111/j.1365-2672.2008.03918.x

[B73] Isvanovics V. Shafik H. Presing M. Juhos Z. (2000). Growth and phosphate uptake kinetics of the cyanobacterium, *Cylindrospermopsis raciborskii* (Cyanophyceae) in throughflow cultures. *Freshw. Biol.* 43 257–275. 10.1046/j.1365-2427.2000.00549.x

[B74] James K. Crowley J. Hamilton B. Lehane M. Skulberg O. Furey A. (2005). Anatoxins and degradation products, determined using hybrid quadrupole time-of-flight and quadrupole ion-trap mass spectrometry: Forensic investigations of cyanobacterial neurotoxin poisoning. *Rapid Commun. Mass Sp.* 19 1167–1175. 10.1002/rcm.1894 15816010

[B75] Jensen J. Cox R. (1983). Direct measurements of steady-state kinetics of cyanobacterial N2 uptake by membrane-leak mass spectrometry and comparisons between nitrogen fixation and acetylene reduction. *Appl. Environ. Microb.* 45 1331–1337. 10.1128/aem.45.4.1331-1337.1983 16346272 PMC242459

[B76] John N. Baker L. Ansell B. Newham S. Crosbie N. Jex A. (2019). First report of anatoxin-a producing cyanobacteria in Australia illustrates need to regularly update monitoring strategies in a shifting global distribution. *Sci. Rep.* 9:10894. 10.1038/s41598-019-46945-8 31350418 PMC6659621

[B77] Jones M. (1984). Nitrate reduction by shaking with cadmium: Alternative to cadmium columns. *Water Res.* 18 643–646. 10.1016/0043-1354(84)90215-X

[B78] Kanehisa M. Goto S. Sato Y. Kawashima M. Furumichi M. Tanabe M. (2014). Data, information, knowledge and principle: Back to metabolism in KEGG. *Nucleic Acids Res.* 42 D199–D205. 10.1093/nar/gkt1076 24214961 PMC3965122

[B79] Kearns K. Hunter M. (2001). Toxin-Producing *Anabaena flos-aquae* Induces Settling of *Chlamydomonas reinhardtii*, a Competing Motile Alga. *Microb. Ecol.* 42 80–86. 10.1007/s002480000086 12035083

[B80] Krausfeldt L. Farmer A. Castro Gonzalez H. Zepernick B. Campagna S. Wilhelm S. (2019). Urea is both a carbon and nitrogen source for *Microcystis aeruginosa*: Tracking ^13^łC incorporation at bloom pH conditions. *Front. Microbiol.* 10:1064. 10.3389/fmicb.2019.01064 31164875 PMC6536089

[B81] Lewis W. Levine S. (1984). The light response of nitrogen fixation in Lake Valencia, Venezuela. *Limnol. Oceanogr.* 29 894–900. 10.4319/lo.1984.29.4.0894

[B82] Li X. Dreher T. Li R. (2016). An overview of diversity, occurrence, genetics and toxin production of bloom-forming *Dolichospermum* (*Anabaena*) species. *Harmful Algae* 54 54–68. 10.1016/j.hal.2015.10.015 28073482

[B83] Lilleheil G. Andersen R. Skulberg O. Alexander J. (1997). Effects of a homoanatoxin-a-containing extract from *Oscillatoria formosa* (cyanophyceae/cyanobacteria) on neuromuscular transmission. *Toxicon* 35 1275–1289. 10.1016/S0041-0101(97)00013-5 9278976

[B84] Lin W. Zhao D. Luo J. (2018). Distribution of alkaline phosphatase genes in cyanobacteria and the role of alkaline phosphatase on the acquisition of phosphorus from dissolved organic phosphorus for cyanobacterial growth. *J. Appl. Phycol.* 30 839–850. 10.1007/s10811-017-1267-3

[B85] Lu J. Zhu B. Struewing I. Xu N. Duan S. (2019). Nitrogen–phosphorus- associated metabolic activities during the development of a cyanobacterial bloom revealed by metatranscriptomics. *Sci. Rep.* 9:2480. 10.1038/s41598-019-38481-2 30792397 PMC6385219

[B86] Martin−Níeto J. Herrero A. Flores E. (1991). Control of nitrogenase mRNA levels by products of nitrate assimilation in the cyanobacterium *Anabaena* sp. Strain PCC 7120. *Plant Physiol.* 97 825–828. 10.1104/pp.97.2.825 16668475 PMC1081083

[B87] Meeks J. Wycoff K. Chapman J. Enderlin C. (1983). Regulation of expression of nitrate and dinitrogen assimilation by *Anabaena* Species. *Appl. Environ. Microb.* 45 1351–1359. 10.1128/aem.45.4.1351-1359.1983 16346274 PMC242462

[B88] Mekonnen A. Prasanna R. Kaushik B. (2002). Response of *Anabaena* species to different nitrogen sources. *Acta. Biol. Hung.* 53 367–380. 10.1556/abiol.53.2002.3.13 12371616

[B89] Mickelson J. Davis E. Tischer R. (1967). The effect of various nitrogen sources upon heterocyst formation in *Anabaena Flos-Aquae* A-37. *J. Exp. Bot.* 18 397–405. 10.1093/jxb/18.3.397

[B90] Mishra A. (1997). Regulation of cellular constituents, heterocyst development, photosynthetic O2 evolution and enzyme activites of *Anabaena* sp, PCC 7120 by nitrogen sources. *Cytobios* 89 173–182. 10.1128/JB.185.23.6995-7000.2003 14617665 PMC262716

[B91] Mitschke J. Vioque A. Haas F. Hess W. Muro-Pastor M. (2011). Dynamics of transcriptional start site selection during nitrogen stress-induced cell differentiation in *Anabaena* sp. PCC7120. *PNAS* 108 20130–20135. 10.1073/pnas.1112724108 22135468 PMC3250118

[B92] Mobley H. Hausinger R. (1989). Microbial ureases: Significance, regulation, and molecular characterization. *Microbiol. Rev.* 53 85–108. 10.1128/mr.53.1.85-108.1989 2651866 PMC372718

[B93] Molot L. (2017). The effectiveness of cyanobacteria nitrogen fixation: Review of bench top and pilot scale nitrogen removal studies and implications for nitrogen removal programs. *Environ. Rev.* 25 1–16. 10.1139/er-2016-0107

[B94] Montesinos M. Muro-Pastor A. Herrero A. Flores E. (1998). Ammonium/methylammonium permeases of a cyanobacterium. *J. Biol. Chem.* 273 31463–31470. 10.1074/jbc.273.47.31463 9813059

[B95] Muro-Pastor M. Reyes J. Florencio F. (2001). Cyanobacteria Perceive Nitrogen Status by Sensing intracellular 2-oxoglutarate levels. *J. Biol. Chem.* 41 38320–38328. 10.1074/jbc.M105297200 11479309

[B96] Muro-Pastor M. Reyes J. Florencio F. (2005). Ammonium assimilation in cyanobacteria. *Photosynth. Res.* 83 135–150. 10.1007/s11120-004-2082-7 16143848

[B97] Ninfa A. Magasanik B. (1986). Covalent modification of the glnG product, NRI, by the glnL product, NRII, regulates the transcription of the glnALG operon in *Escherichia coli*. *PNAS* 83 5909–5913. 10.1073/pnas.83.16.5909 2874557 PMC386406

[B98] Nonaka A. Yamamoto H. Kamiya N. Kotani H. Yamakawa H. Tsujimoto R. (2019). Accessory proteins of the nitrogenase assembly, NifW, NifX/NafY, and NifZ, are essential for diazotrophic growth in the nonheterocystous cyanobacterium *Leptolyngbya boryana*. *Front. Microbiol.* 10:495. 10.3389/fmicb.2019.00495 30930880 PMC6428710

[B99] Nurk S. Bankevich A. Antipov D. Gurevich A. Korobeynikov A. Lapidus A. (2013). Assembling single-cell genomes and mini-metagenomes from chimeric mda products. *J. Comput. Biol.* 20 714–737. 10.1089/cmb.2013.0084 24093227 PMC3791033

[B100] Okuhara H. Matsumura T. Fujita Y. Hase T. (1999). Cloning and inactivation of genes encoding ferredoxin- and nadh-dependent glutamate synthases in the cyanobacterium plectonema boryanum. imbalances in nitrogen and carbon assimilations caused by deficiency of the ferredoxin-dependent enzyme. *Plant Physiol.* 120 33–42. 10.1104/pp.120.1.33 10318681 PMC59265

[B101] Olofsson M. Suikkanen S. Kobos J. Wasmund N. Karlson B. (2020). Basin-specific changes in filamentous cyanobacteria community composition across four decades in the Baltic Sea. *Harmful Algae* 91:101685. 10.1016/j.hal.2019.101685 32057344

[B102] O’Neil J. Davis T. Burford M. Gobler C. (2012). The rise of harmful cyanobacteria blooms: The potential roles of eutrophication and climate change. *Harmful Algae* 14 313–334. 10.1016/j.hal.2011.10.027

[B103] Österholm J. Popin R. Fewer D. Sivonen K. (2020). Phylogenomic analysis of secondary metabolism in the toxic cyanobacterial Genera *Anabaena*, *Dolichospermum* and *Aphanizomenon*. *Toxins* 12:248. 10.3390/toxins12040248 32290496 PMC7232259

[B104] Paerl H. (2017). The cyanobacterial nitrogen fixation paradox in natural waters. *F1000Res* 6:244. 10.12688/f1000research.10603.1 28357051 PMC5345769

[B105] Paerl H. Scott J. McCarthy M. Newell S. Gardner W. Havens K. (2016). It takes two to tango: When and where dual nutrient (N & P) reductions are needed to protect lakes and downstream ecosystems. *Environ. Sci. Technol.* 50 10805–10813. 10.1021/acs.est.6b02575 27667268

[B106] Paerl H. Xu H. Hall N. Zhu G. Qin B. Wu Y. (2014). Controlling cyanobacterial blooms in hypertrophic Lake Taihu, China: Will nitrogen reductions cause replacement of non−N2 fixing by N2 fixing taxa? *PLoS One* 9:e113123. 10.1371/journal.pone.0113123 25405474 PMC4236137

[B107] Parkhill J. Maillet G. Cullen J. (2001). Fluorescence-based maximal quantum yield for PSII as a diagnostic of nutrient stress. *J. Phycol.* 37 517–529. 10.1046/j.1529-8817.2001.037004517.x

[B108] Parsons T. Maita Y. Lalli C. (1984). *A manual of chemical and biological methods for seawater analysis.* Oxford: Pergamon Press.

[B109] Paul A. Achterberg E. Bach L. Boxhammer T. Czerny J. Haunost M. (2016). No observed effect of ocean acidification on nitrogen biogeochemistry in a summer Baltic Sea plankton community. *Biogeosciences* 13 3901–3913. 10.5194/bg-13-3901-2016

[B110] Paz-Yepes J. Flores E. Herrero A. (2009). Expression and mutational analysis of the glnB genomic region in the heterocyst-forming cyanobacterium *Anabaena* sp. Strain PCC 7120. *J. Bacteriol.* 191 2353–2361. 10.1128/JB.01381-08 19181812 PMC2655514

[B111] Paz-Yepes J. Merino-Puerto V. Herrero A. Flores E. (2008). The amt gene cluster of the heterocyst-forming cyanobacterium *Anabaena* sp. Strain PCC 7120. *J. Bacteriol.* 190 6534–6539. 10.1128/JB.00613-08 18689479 PMC2566009

[B112] Picossi S. Flores E. Herrero A. (2014). ChIP analysis unravels an exceptionally wide distribution of DNA binding sites for the NtcA transcription factor in a heterocyst-forming cyanobacterium. *BMC Genom.* 15:22. 10.1186/1471-2164-15-22 24417914 PMC3898017

[B113] Pimentel H. Bray N. Puente S. Melsted P. Pachter L. (2017). Differential analysis of rna-seq incorporating quantification uncertainty. *Nat. Methods* 14 687–690. 10.1038/nmeth.4324 28581496

[B114] Presing M. Herodek S. Voros L. Kobor I. (1996). Nitrogen fixation, ammonium and nitrate uptake during a bloom of *Cylindrospermopsis raciborskii* in Lake Balaton. *Arch. Hydrobiol.* 136 553–562. 10.1127/archiv-hydrobiol/136/1996/553

[B115] Qian Z. Ma J. Sun C. Li Z. Xian Q. Gong T. (2017). Using stable isotope labeling to study the nitrogen metabolism in *Anabaena flos-aquae* growth and anatoxin biosynthesis. *Water Res.* 127 223–229. 10.1016/j.watres.2017.09.060 29055827

[B116] Ramos J. Guerrero M. (1983). Involvement of ammonium metabolism in the nitrate inhibition of nitrogen fixation in *Anabaena* sp. strain ATCC 33047. *Arch. Microbiol.* 136 81–83. 10.1007/BF00404777

[B117] Rantala-Ylinen A. Kana S. Wang H. Rouhiainen L. Wahlsten M. Rizzi E. (2011). Anatoxin-a synthetase gene cluster of the cyanobacterium *Anabaena* sp. strain 37 and molecular methods to detect potential producers. *Appl. Environ. Microb.* 77 7271–7278. 10.1128/AEM.06022-11 21873484 PMC3194866

[B118] Rapala J. Sivonen K. (1998). Assessment of environmental conditions that favor hepatotoxic and neurotoxic *Anabaena* spp. strains cultured under light limitation at different temperatures. *Microb. Ecol.* 36 181–192. 10.1007/s002489900105 9688780

[B119] Rapala J. Sivonen K. Luukkainen R. Niemela S. (1993). Anatoxin-a concentration in *Anabaena* and *Aphanizomenon* under different environmental conditions and comparison of growth by toxic and non-toxic *Anabaena*-strains - a laboratory study. *J. Appl. Phycol.* 5 581–591. 10.1007/BF02184637

[B120] Redfield A. (1958). The biological control of chemical factors in the environment. *Am. Sci*. 46, 205–221. 24545739

[B121] Reitzer L. Magasanik B. (1985). Expression of glnA in *Escherichia coli* is regulated at tandem promoters. *PNAS* 82 1979–1983. 10.1073/pnas.82.7.1979 2858855 PMC397465

[B122] Rhee G. Lederman T. (1983). Effects of nitrogen sources on P-limited Growth of *Anabaena Flos-aquae*. *J. Phycol.* 19 179–185. 10.1111/j.0022-3646.1983.00179.x

[B123] Rouhiainen L. Sivonen K. Buikema W. Haselkorn R. (1995). Characterization of toxin-producing cyanobacteria by using an oligonucleotide probe containing a tandemly repeated heptamer. *J. Bacteriol.* 177 6021–6026. 10.1128/jb.177.20.6021-6026.1995 7592362 PMC177437

[B124] Sakamoto T. Delgaizo V. Bryant D. (1998). Growth on urea can trigger death and peroxidation of the cyanobacterium *Synechococcus* sp. Strain PCC 7002. *Appl. Environ. Microbiol.* 64, 2361–2366. 10.1128/aem.64.7.2361-2366.1998 9647800 PMC106396

[B125] Salmaso N. Capelli C. Shams S. Cerasino L. (2015). Expansion of bloom-forming *Dolichospermum lemmermannii* (Nostocales, Cyanobacteria) to the deep lakes south of the Alps: Colonization patterns, driving forces and implications for water use. *Harmful Algae* 50 76–87. 10.1016/j.hal.2015.09.008

[B126] Sanchez J. Otero P. Alfonso A. Ramos V. Vasconcelos V. Aráoz R. (2014). Detection of anatoxin-a and three analogs in *Anabaena* spp. cultures: New fluorescence polarization assay and toxin profile by LC-MS/MS. *Toxins* 6 402–415. 10.3390/toxins6020402 24469431 PMC3942742

[B127] Sanz A. Moreno-Vivian C. Maldonado J. Gonzalez-Fontes A. (1995). Effect of a constant supply of different nitrogen sources on protein and carbohydrate content and enzyme activities of *Anabaena variabilis* cells. *Physiol. Plant* 95 39–44. 10.1111/j.1399-3054.1995.tb00805.x

[B128] Sanz-Luque E. Bhaya D. Grossman A. (2020). Polyphosphate: A multifunctional metabolite in cyanobacteria and algae. *Front. Plant Sci.* 11:938. 10.3389/fpls.2020.00938 32670331 PMC7332688

[B129] Schindler D. Hecky R. Findlay D. Stainton M. Parker B. Paterson M. (2008). Eutrophication of lakes cannot be controlled by reducing nitrogen input: Results of a 37-year whole-ecosystem experiment. *Proc. Natl. Acad. Sci. U. S. A.* 105 11254–11258. 10.1073/pnas.0805108105 18667696 PMC2491484

[B130] Scott J. McCarthy M. (2010). Nitrogen fixation may not balance the nitrogen pool in lakes over timescales relevant to eutrophication management. *Limnol. Oceanogr.* 55 1265–1270. 10.4319/lo.2010.55.3.1265

[B131] Seeman T. (2014). Prokka: Rapid prokaryotic genome annotation. *Bioinformatics* 30 2068–2069. 10.1093/bioinformatics/btu153 24642063

[B132] Serrano A. Rivas J. Losada M. (1981). Nitrate and nitrite as ‘in vivo’ quenchers of chlorophyll fluorescence in blue-green algae. *Photosynth. Res.* 2 175–184. 10.1007/BF00032356 24470230

[B133] Shatwell T. Köhler J. (2018). Decreased nitrogen loading controls summer cyanobacterial blooms without promoting nitrogen-fixing taxa: Long-term response of a shallow lake. *Limnol. Oceanogr.* 64, 1–13. 10.1002/lno.11002

[B134] Simis S. Huot Y. Babin M. Seppala J. Metsamaa L. (2012). Optimization of variable fluorescence measurements of phytoplankton communities with cyanobacteria. *Photosynth. Res.* 112 13–30. 10.1007/s11120-012-9729-6 22403036 PMC3324691

[B135] Smith V. (1983). Low nitrogen to phosphorus ratios favor dominance by blue-green algae in lake phytoplankton. *Science* 221 669–671. 10.1126/science.221.4611.669 17787737

[B136] Smith V. (2016). Effects of eutrophication on maximum algal biomass in lake and river ecosystems. *Inland Waters* 6 147–154. 10.5268/IW-6.2.937147ArticleEffects10.5268/IW-6.2.937Val

[B137] Spivak C. Witkop B. Albuquerque E. (1980). Anatoxin-a: A novel, potent agonist at the nicotinic receptor. *Mol. Pharmacol.* 18 384–394. 6970328

[B138] Stacey G. Tabita F. Van Baalen C. (1977). Nitrogen and Ammonia Assimilation in the Cyanobacteria: Purification of Glutamine Synthetase from *Anabaena* sp. Strain CA. *J. Bacteriol.* 132 596–603. 10.1128/jb.132.2.596-603.1977 21167 PMC221901

[B139] Stainer R. Kunisawa R. Mandel M. Cohen-Bazire G. (1971). Purification and properties of unicellular blue-green algae (order Chroococcales). *Bacteriol. Rev.* 35 171–205. 10.1128/br.35.2.171-205.1971 4998365 PMC378380

[B140] Stucken K. John U. Cembella A. Soto-Liebe K. Vasquez M. (2014). Impact of nitrogen sources on gene expression and toxin production in the diazotroph *Cylindrospermopsis raciborskii* CS-505 and Non-Diazotroph *Raphidiopsis brookii* D9. *Toxins* 6 1896–1915. 10.3390/toxins6061896 24956074 PMC4073136

[B141] Suzuki S. Kakuta M. Ishida T. Akiyama Y. (2014). GHOSTX: An improved sequence homology search algorithm using a query suffix array and a database suffix array. *PLoS One* 9:e103833. 10.1371/journal.pone.0103833 25099887 PMC4123905

[B142] Tao S. Wang S. Song L. Gan N. (2020). Understanding the differences in the growth and toxin production of anatoxin-producing *Cuspidothrix issatschenkoi* cultured with inorganic and organic N sources from a new perspective: Carbon/nitrogen metabolic balance. *Toxins* 12:724. 10.3390/toxins12110724 33228063 PMC7699347

[B143] Teikari J. Osterholm J. Kopf M. Battchikova N. Wahlsten M. Aro E. M. (2015). Transcriptomic and proteomic profiling of *anabaena* sp. strain 90 under inorganic phosphorus stress. *Appl. Environ. Microbiol.* 81 5212–5222. 10.1128/AEM.01062-15 26025890 PMC4495225

[B144] Toporowska M. Pawlik-Skowronska B. Kalinowska R. (2014). Accumulation and effects of cyanobacterial microcystins and anatoxin-a on benthic larvae of *Chironomus* spp. (Diptera: Chironomidae). *Eur. J. Entomol.* 111 83–90. 10.14411/eje.2014.010

[B145] Toporowska M. Pawlik-Skowronska B. Kalinowska R. (2016). Mass development of diazotrophic cyanobacteria (Nostocales) and production of neurotoxic anatoxin-a in a *Planktothrix* (Oscillatoriales) dominated temperate lake. *Water Air Soil Pollut.* 227 321. 10.1007/s11270-016-3004-y 27546924 PMC4980406

[B146] Tsinoremas N. Castets A. Harrison M. Allen J. Tandeau de Marsac N. (1991). Photosynthetic electron transport controls nitrogen assimilation in cyanobacteria by means of posttranslational modification of the glnB gene product. *PNAS* 88 4565–4569. 10.1073/pnas.88.11.4565 1905010 PMC51705

[B147] Ugalde R. Imperial J. Shah V. Brill W. (1984). Biosynthesis of iron-molybdenum cofactor in the absence of nitrogenase. *J. Bacteriol.* 159 888–893. 10.1128/jb.159.3.888-893.1984 6384184 PMC215742

[B148] Valladares A. Flores E. Herrero A. (2008). Transcription activation by NtcA and 2-oxoglutarate of three genes involved in heterocyst differentiation in the cyanobacterium *Anabaena* sp. Strain PCC 7120. *J. Bacteriol.* 190 6126–6133. 10.1128/JB.00787-08 18658268 PMC2546789

[B149] Valladares A. Montesinos M. Herrero A. Flores E. (2002). An ABC-type, high-affinity urea permease identified in cyanobacteria. *Mol. Microbiol.* 43 703–715. 10.1046/j.1365-2958.2002.02778.x 11929526

[B150] Van de Waal D. B. Smith V. H. Declerck S. A. Stam E. C. Elser J. J. (2014). Stoichiometric regulation of phytoplankton toxins. *Ecol. Lett.* 17 736–742. 10.1111/ele.12280 24712512

[B151] Veaudor T. Cassier-Chauvat C. Chauvat F. (2019). Genomics of urea transport and catabolism in cyanobacteria: Biotechnological implications. *Front. Microbiol.* 10:2052. 10.3389/fmicb.2019.02052 31551986 PMC6737895

[B152] Velzeboer R. Baker P. Rositano J. (2001). Saxitoxins associated with the growth of the cyanobacterium *Anabaena circinalis* (Nostocales, Cyanophyta) under varying sources and concentrations of nitrogen. *Phycologia* 40 305–312. 10.2216/i0031-8884-40-3-305.1

[B153] Voß B. Bolhuis H. Fewer D. Kopf M. Moke F. Haas F. (2013). Insights into the physiology and ecology of the brackish-water-adapted cyanobacterium *Nodularia spumigena* CCY9414 based on a genome-transcriptome analysis. *PLoS One* 8:e60224. 10.1371/journal.pone.0060224 23555932 PMC3610870

[B154] Wacklin P. Hoffmann L. Komárek J. (2009). Nomenclatural validation of the genetically revised cyanobacterial genus *Dolichospermum* (Ralfs ex Bornet et Flahault) comb. nova. *Fottea* 9 59–64. 10.5507/fot.2009.005

[B155] Walker M. van der Donk W. (2016). The many roles of glutamate in metabolism. *J. Ind. Microbiol. Biotechnol.* 43 419–430. 10.1007/s10295-015-1665-y 26323613 PMC4753154

[B156] Wan L. Chen X. Deng Q. Yang L. Li X. ZHang J. (2019). Phosphorus strategy in bloom-forming cyanobacteria (*Dolichospermum* and *Microcystis*) and its role in their succession. *Harmful Algae* 84 46–55. 10.1016/j.hal.2019.02.007 31128812

[B157] Wang Z. Akbar S. Sun Y. Gu L. Zhang L. Lyu K. (2021). Cyanobacterial dominance and succession: Factors, mechanisms, predictions, and managements. *J. Environ. Manage.* 297:113281. 10.1016/j.jenvman.2021.113281 34274765

[B158] Wannicke N. Herrmann A. Gehringer M. (2021). Atmospheric CO2 availability induces varying responses in net photosynthesis, toxin production and N2 fixation rates in heterocystous filamentous Cyanobacteria (*Nostoc* and *Nodularia*). *Aquat. Sci.* 83:33. 10.1007/s00027-021-00788-6

[B159] Willis A. Chuang A. W. Burford M. A. (2016). Nitrogen fixation by the diazotroph *Cylindrospermopsis raciborskii* (Cyanophyceae). *J. Phycol.* 52 854–862. 27440068 10.1111/jpy.12451

[B160] Wolk C. Ernst A. Elhai J. (1994). “Heterocyst Metabolism and Development,” in *The molecular biology of cyanobacteria. Advances in photosynthesis*, ed. Bryant D. (Dordrecht: Springer), 769–823.

[B161] Wolk C. Thomas J. Shaffer P. Austin S. Galonsky A. (1976). Pathway of nitrogen metabolism after fixation of ^13^łN-labeled nitrogen gas by the cyanobacterium, *Anabaena cylindrica*. *J. Biol. Chem.* 251 5027–5034. 10.1016/S0021-9258(17)33216-7 821946

[B162] Wu Z. Zeng B. Li R. Song L. (2012). Physiological regulation of *Cylindrospermopsis raciborskii* (Nostocales, Cyanobacteria) in response to inorganic phosphorus limitation. *Harmful Algae* 15 53–58. 10.1016/j.hal.2011.11.005

[B163] Wurtsbaugh W. Horne A. (1983). Iron in Eutrophic Clear Lake, California: Its Importance for Algal Nitrogen Fixation and Growth. *Can. J. Fish Aquat. Sci.* 40 1419–1429. 10.1139/f83-164

[B164] Zhao M. Jiang Y. He Y. Chen Y. Teng Y. Chen Y. (2010). Structural basis for the allosteric control of the global transcription factor NtcA by the nitrogen starvation signal 2-oxoglutarate. *Proc. Natl. Acad. Sci. U. S. A.* 107 12487–12492. 10.1073/pnas.1001556107 20616047 PMC2906582

[B165] Zulkefli N. Hwang S. (2020). Heterocyst development and diazotrophic growth of *Anabaena variabilis* under different nitrogen availability. *Life* 10:279. 10.3390/life10110279 33202779 PMC7696877

