# Supplementary material for: Nitrogen and phosphorus significantly alter growth, nitrogen fixation, anatoxin-a content, and the transcriptome of the bloom-forming cyanobacterium, Dolichospermum
Source: Front Microbiol. 2022 Sep 7;13:955032. doi: 10.3389/fmicb.2022.955032 (PMC9490380; doi:10.3389/fmicb.2022.955032)
Supplement: Supplementary file 1 [file Table_1.docx]

| Treatment | Day 3 NO_3_^-^ | Day 3 NH_3_ | Day 6 NO_3_^-^ | Day 6 NH_3_ | Day 7 NO_3_^-^ | Day 7 NH_3_ |
| --- | --- | --- | --- | --- | --- | --- |
| -N+P (control) | BDL | 2.24 ± 1.26 | 0.88 ± 4.74 | 2.80 ± 1.86 | 2.69 ± 5.47 | 1.12 ± 0.51 |
| NH_4_^+^+P | BDL | 2.66 ± 0.95 | BDL | 164 ± 20.1 * (A) | BDL | 214 ± 21.6 * (A) |
| Urea+P | 0.15 ± 1.12 | 2.31 ± 0.40 | BDL | 88.9 ± 7.49 * (B) | BDL | 123 ± 9.93 * (B) |
| NO_3_^-^+P | 25.7 ± 5.59 * | 1.48 ± 0.35 | 376 ± 23.6 * | 1.57 ± 0.85 | 640 ± 40.7 * | 1.01 ± 0.79 |
| -N-P | 0.36 ± 1.32 | 2.08 ± 1.13 | BDL | 2.09 ± 0.43 | 2.48 ± 5.02 | 0.86 ± 0.27 |

Supplemental Table 1: Dissolved fixed nitrogen concentrations (*µ*M) on days 3, 6, and 7. ± = standard deviation. Asterisks (Two-way ANOVA) and letters (Tukey’s HSD post-hoc) represent significant (*p*<0.05) differences between experimental groups. BDL = below detection limit (NO_3_^-^ detection limit = 1.47x10^-2^ *µ*M; NH_3_ = 9.19x10^-3^ *µ*M).
