# Supplementary figures and images for: Nitrogen and phosphorus significantly alter growth, nitrogen fixation, anatoxin-a content, and the transcriptome of the bloom-forming cyanobacterium, Dolichospermum
Source: Front Microbiol. 2022 Sep 7;13:955032. doi: 10.3389/fmicb.2022.955032 (PMC9490380; doi:10.3389/fmicb.2022.955032)

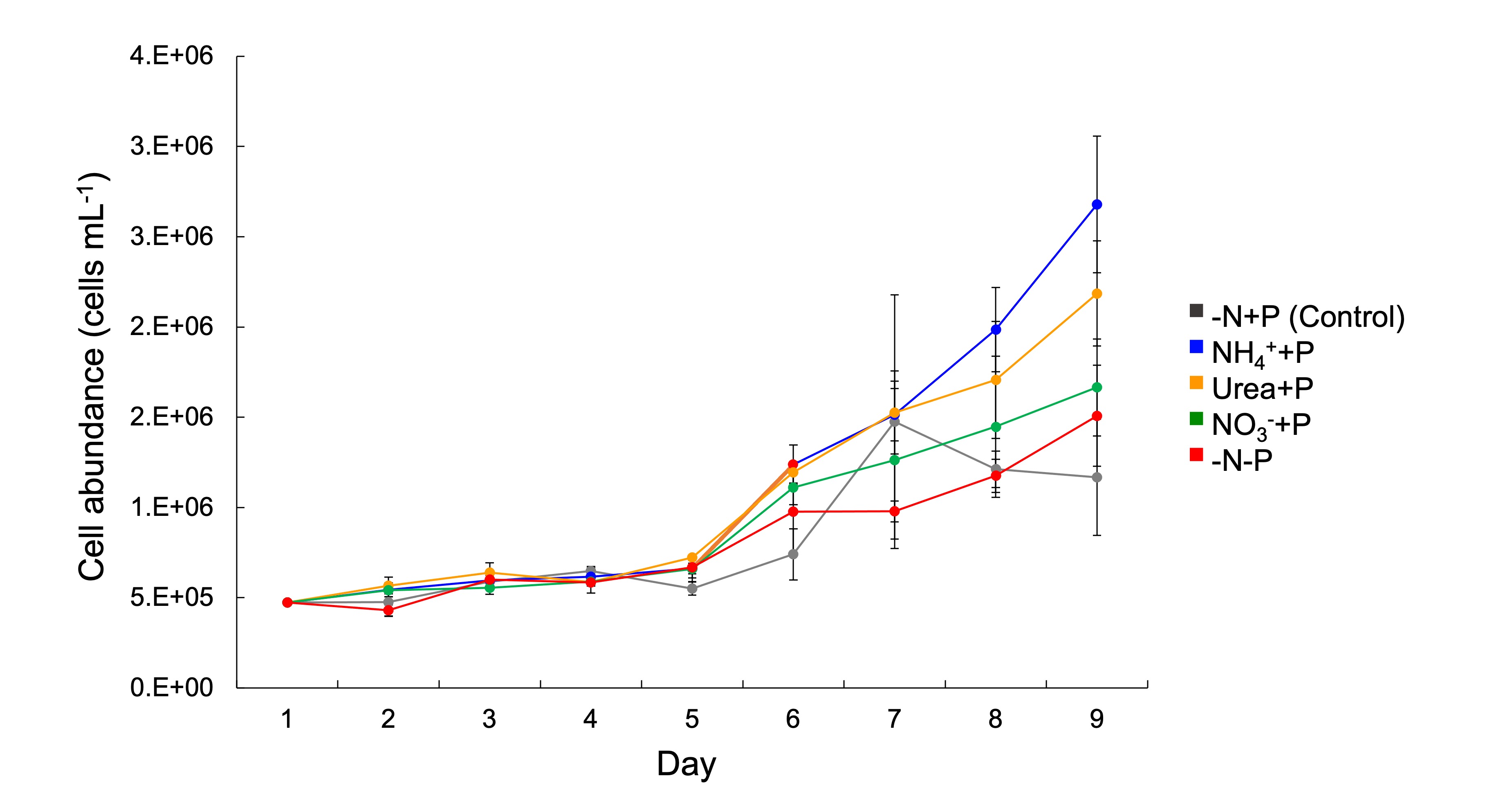

Supplement: Supplementary Figure 1 — Cell densities for all treatments during the experiment, from day 1 to the final time point (day 9). Error bars represent standard deviation. [file Image_1.JPEG]

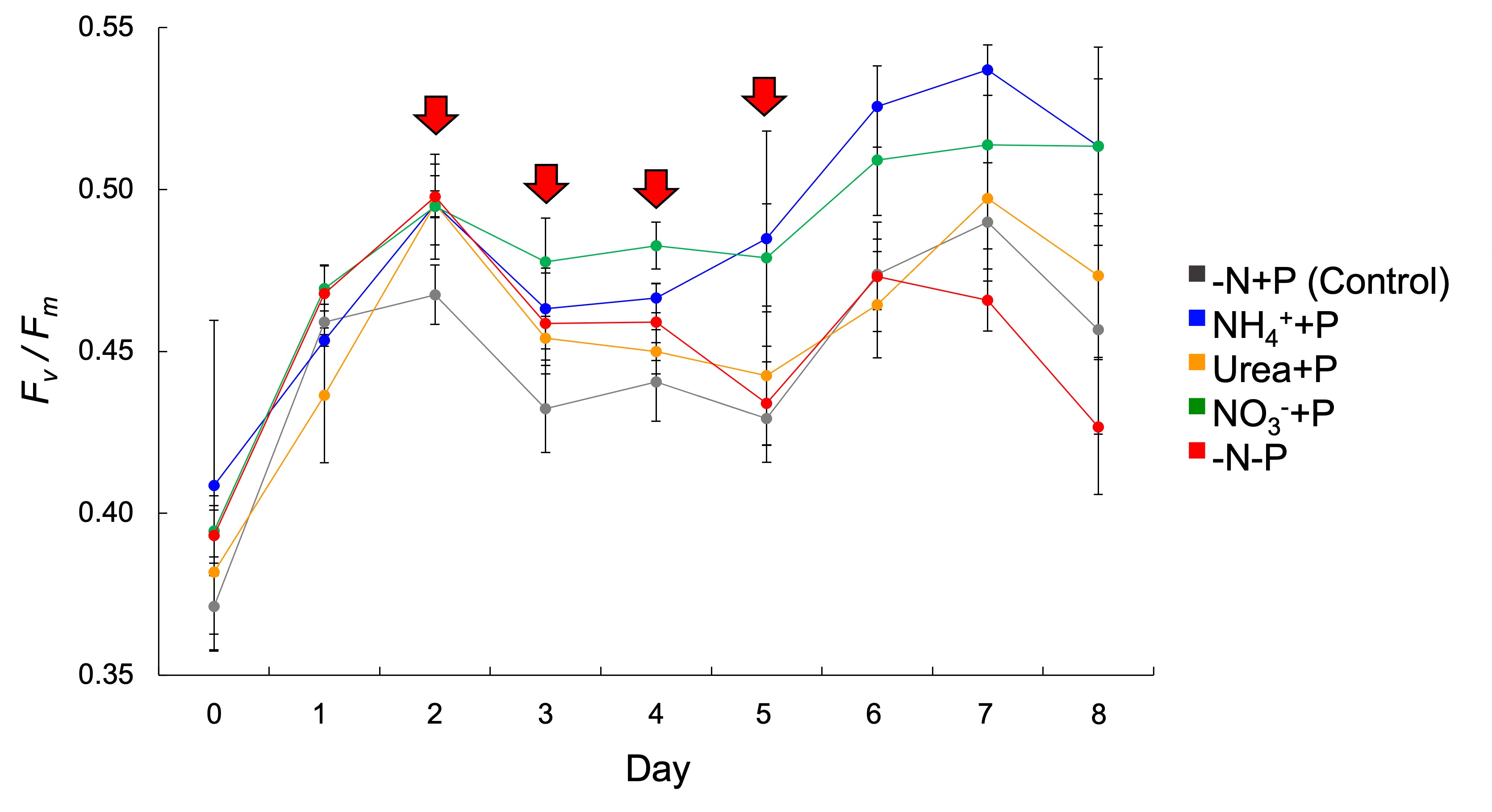

Supplement: Supplementary Figure 2 — Photosystem II photosynthetic efficiency (Fv/Fm) for all treatments during the experiment. Error bars represent standard deviation. Red arrows represent days when fixed N species (NH4Cl, Urea, and NaNO3) were added to fixed N-replete treatments. [file Image_2.JPEG]

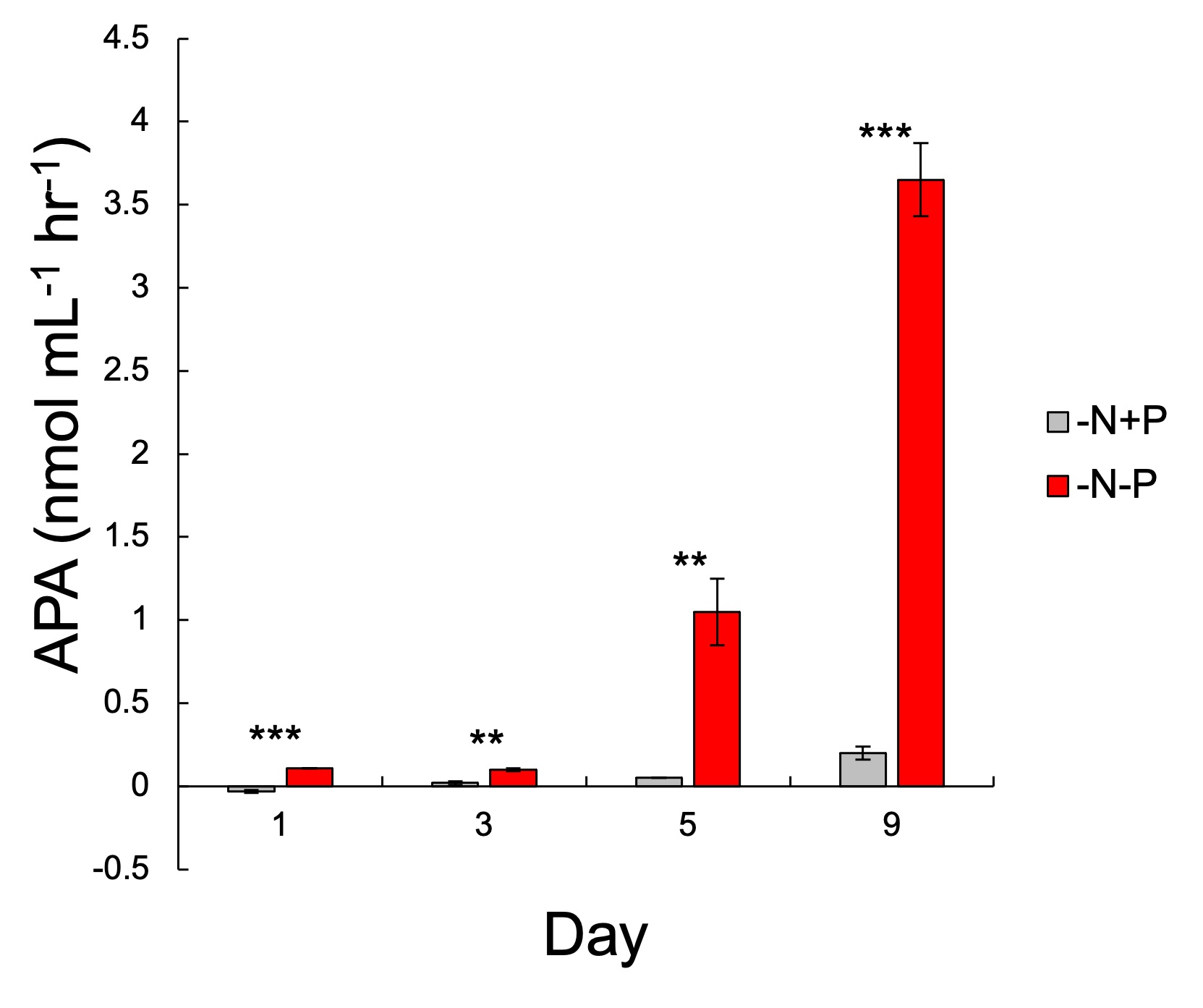

Supplement: Supplementary Figure 3 — Alkaline phosphatase activity (nmol mL–1 hr–1) as a function of volume and time in treatments deprived on nitrogen and either with or without phosphorus on days 1, 3, 5, and 9. Error bars represent standard error. Number of asterisks correspond to degree of significance (p < 0.05 = *, 0.01 = **, 0.001 = ***), determined via Student’s paired t-test. [file Image_3.JPEG]
